# Macrophage lipophagy in atherosclerosis: from molecular mechanisms to traditional Chinese medicine interventions

**DOI:** 10.3389/fphar.2026.1916672

**Published:** 2026-10-15

**Authors:** Ying Hu, Shuo Yuan, Xingjuan Chen, Yu Wen, Fang Liu, Xuewei Dong, Mengxuan Cui, Na Zhao, Hua Dong

**Affiliations:** 1 First Teaching Hospital of Tianjin University of Traditional Chinese Medicine, National Clinical Research Center for Chinese Medicine, Tianjin, China; 2 Children’s Hospital Affiliated to Zhengzhou University, Henan Children’s Hospital, Zhengzhou Children’s Hospital, Zhengzhou, China; 3 Guang’anmen Hospital of Chinese Academy of Traditional Chinese Medicine, Beijing, China; 4 Tianjin University of Traditional Chinese Medicine, Tianjin, China

**Keywords:** atherosclerosis, autophagy, lipid homeostasis, lipophagy, macrophages, traditional Chinese medicine (TCM)

## Abstract

Macrophage lipophagy, the selective autophagic degradation of intracellular lipid droplets, plays a central role in maintaining lipid homeostasis and has emerged as a promising therapeutic target for atherosclerosis (AS). This review systematically synthesizes the classification, molecular mechanisms, and key signaling networks of macrophage lipophagy. Moreover, recent evidence on the anti-AS effects of Traditional Chinese Medicine (TCM) mediated by the regulation of macrophage lipophagy has also been summarized and evaluated, including active metabolites, medicinal plants, herbal formulas and commercial Chinese polyherbal preparations. Despite robust preclinical evidence, challenges remain. Significant efforts are still required to facilitate clinical translation and offer novel comprehensive strategies for the prevention and management of AS.

## 1 Introduction

Atherosclerosis (AS) is an inflammatory disease characterized by the accumulation of lipids within the walls of large arteries (Döring et al., 2024). Epidemiological data reveal a high prevalence of atherosclerotic plaques. In southeastern China, over 90% of the elderly in community settings have atherosclerotic plaques, and approximately 80% of them show plaques in multiple vascular regions (Pan et al., 2022). The rupture of vulnerable atherosclerotic plaques and subsequent local thrombosis constitute key pathological mechanisms underlying ischemic cardio-cerebrovascular events, such as stroke (Prochilo et al., 2024) and coronary heart disease (Tsao et al., 2023). Advances have been made in the management of AS, including the use of statins, ezetimibe, and PCSK9 inhibitors to lower cholesterol levels and alleviate disease progression (Doi et al., 2023; Libby, 2021). However, clinical utility is partly limited by statin intolerance and potential long-term adverse effects such as myalgia, hepatic injury, and new-onset diabetes (National Society of Cardiometabolic Medicine, 2024; Visseren et al., 2021). Emerging therapeutic strategies, including gene editing, siRNA-targeted therapy (Ray et al., 2023), and nanodrug delivery systems (Fu et al., 2022), have shown promising potential. Nevertheless, these approaches remain largely in developmental stages, facing significant challenges such as precise and reproducible synthesis, which hinder their widespread clinical application. With increasing life expectancy and an aging global population, the morbidity and mortality of AS-related cardio-cerebrovascular diseases are expected to rise steadily (Woodruff et al., 2024). Therefore, the exploration of novel therapeutic targets and intervention strategies remains an urgent and necessary endeavor.

In the intricate pathological process of AS, macrophages serving as key immune effector cells are closely linked to disease progression. Lipophagy is a selective form of autophagy, which degrades intracellular lipid droplets (LDs) via the autophagy-lysosomal pathway (Liu and Czaja, 2013). By activating lipophagy related molecules, macrophages selectively recognize and degrade cholesterol and triglycerides within LDs, clear damaged organelles, and attenuate foam cell formation (Zhang et al., 2022; Yuan et al., 2024). Furthermore, activation of lipophagy promotes polarization of macrophages toward an anti-inflammatory M2 phenotype, which inhibits the release of inflammatory factors (Liu T. et al., 2021). Additionally, it can enhance cholesterol efflux (Chen et al., 2024) and reduce oxidative stress (Zhu L. et al., 2024), thus alleviating the pathological progression of AS. When macrophage lipophagy is dysfunctional, lysosomal lipid clearance and cholesterol efflux decline, leading to intracellular accumulation of excess lipids and formation of foam cells (Laval and Ouimet, 2023). Thus, targeted activation of the macrophage lipophagy pathway is an important therapeutic strategy to inhibit foam cell formation and prevent AS.

Traditional Chinese Medicine (TCM) has shown effectiveness in the prevention and treatment of AS, including herbal formulas, commercial Chinese polyherbal preparations (CCPPs), TCM injections, and herbal extracts (Liu et al., 2024). Studies have reported that herbal active compounds can ameliorate AS by acting on multiple signaling pathways, and, importantly, modulate lipophagy in macrophages (Zhang M. et al., 2024; Cui et al., 2024). While reviews (Shao et al., 2016; Liu et al., 2020; Zahid et al., 2021; Laval and Ouimet, 2023; Nan et al., 2026) have discussed the relationship between macrophages and AS or the role of lipophagy in AS, articles focusing on macrophage lipophagy and the effects of TCM remain scarce. Focusing on monocyte-derived macrophages recruited into the arterial intima during atherogenesis, this review summarized the mechanisms underlying macrophage lipophagy in AS and elucidated the therapeutic potential of TCM agents, offering insights for future research and drug development.

## 2 Search strategy

We searched databases of PubMed, Web of Science, Google Scholar, and CNKI for research published up to December 2025. The search terms included “macrophage,” “lipophagy,” “autophagy,” “atherosclerosis,” “traditional Chinese medicine,” “herb,” and “natural products.” In addition, we searched the names of specific TCM herbs and their metabolites. Original research articles *in vitro* and *in vivo* were retrieved, while non-macrophage cell types without relevance to macrophage lipophagy, studies lacking mechanistic data, and non-TCM substances were excluded. Given the emerging nature of the lipophagy field, the direct experimental evidence is limited; some included studies provide indirect evidence (general autophagy markers in macrophages or LD degradation, etc.) and are noted in Tables 1–4.

**TABLE 1:** Mechanisms of herbal metabolites in regulating lipophagy related pathways against atherosclerosis.

Category	Herbal metabolites	*In vivo* (model; duration; dosage; control)	*In vitro* (model; duration; dosage; control)	Autophagy/lipophagy signaling pathways	Anti-AS mechanisms	References
Flavonoids	Calycosin	ApoE^−/−^ mice; fed with HFD for 16 weeks; 60 mg/kg/d; NC	RAW264.7 cells and peritoneal macrophages; 5 μM; 3-MA	1) Enhance the interaction of KLF2/MLKL; 2) Increase the levels of Beclin1, ATG5, LAMP1 and LAL, and decrease the level of p62.	Inhibit foam cell formation, enhance autophagy, promote cholesterol efflux, and suppress inflammation.	Ma et al. (2022)
​	Luteolin	No	RAW264.7 cells; 24 h; 25 μM; 3-MA	Increase the levels of Beclin1, ABCA1 and LC3II/I ratio.	Activate autophagy to suppress foam cell formation and macrophage apoptosis.	Zhang B. C. et al. (2016)
​	​	No	ANA-1 macrophages; 24 h or 48 h; 0–40 μM; NC	1) Modulate AKT and MAPK signaling pathways; 2) Increase the levels of LC3I, ATG7 and ATG12.	Induce macrophage autophagy and apoptosis.	Liao et al. (2018)
​	Quercetin	ApoE^−/−^ mice; gavage for 12 weeks; 12.5 mg/kg/d; NC	RAW264.7 cells; 24 h; 25 μM; 3-MA and Rap	1) Inhibit MST1 pathway; 2) Increase the levels of autophagy number, LC3II/I ratio and Beclin1, and decrease the expression of Bcl-2, mTOR, P53, P21 and P16.	1) Enhance macrophage autophagy, regulate lipid metabolism; 2) Inhibit the expression of inflammatory factors, lipid accumulation and cell aging.	Cao et al. (2019a), Cao et al. (2019b)
​	Formononetin	macrophage-transgenic zebrafish; 8 days; 20 or 40 μM; NC	RAW264.7 cells; 25 h; 20 or 40 μM; NC	Increase the LC3II/I level, and decrease p62 expression.	Enhance autophagy and polarization of macrophages and inhibit inflammation.	Xu et al. (2025)
Saponins	Geniposide	ApoE^−/−^ mice; gavage for 12 weeks; 50 mg/kg/d; simvastatin (5 mg/kg/d)	RAW264.7 cells; 4 h; 0, 20, 40 and 80 μM; Baf	1) Regulate TREM2/mTOR and AMPK/ULK1 signaling pathway; 2) Increase LC3II/I ratio and Beclin1 levels, and decrease p62 levels.	1) Activate macrophages autophagy and enhance plaque stability; 2) Reduce the levels of blood lipid and blood sugar, and inhibit plaque formation.	Xu et al. (2020)
​	Gypenoside	No	THP-1cells; 12 and 48 h; 5, 10, and 25 μM; Baf	1)Regulate Sirt1-FOXO1 pathway; 2) Increase LC3II/I ratio, and decrease p62 levels.	Promote lysosomal biosynthesis, enhance autophagy and inhibit foam cell formation.	Hui et al. (2021)
​	Ginsenoside Rb1	ApoE^−/−^ mice; gavage for 8 weeks; 10 mg/kg/d NC	No	Increase the levels of LC3II/I ratio and Beclin1, and promote the degradation of p62.	Regulate plasma lipid levels, inhibit inflammatory factors and apoptosis, and enhance autophagy.	Zhou et al. (2018)
​	​	ApoE^−/−^ mice; gavage for 8 weeks; 50 mg/kg/d; NC	peritoneal macrophages; 24 h; 10, 20, 40 and 80 μM; Compound C	1) Promote the expression of LC3II/I ratio and SQSTM1/p62 degradation; 2) Promote AMPK phosphorylation.	Increase the levels of autophagolysosomes and reduce lipid accumulation.	Qiao et al. (2017)
​	Celosin	ApoE^−/−^ mice; gavage for 4 weeks; 10, 30, and 90 mg/kg/d; atorvastatin (2 mg/kg/d)	peritoneal macrophages; 24 h; 50 μM; NC	1) Inhibit the expression of CD36 and SR-A1 while upregulate the expression of ABCA1 and ABCG1; 2) Increased the autophagy specific protein LC3 and Beclin1.	Promote macrophage autophagy, inhibit lipid uptake and foam cell formation, and enhance cholesterol efflux.	Tang et al. (2018)
​	Clematichinenoside AR	No	RAW264.7 cells; 72 h; 3, 10, and 30 μM; Rap and Baf	Increase the expression of LC3II/I, Beclin1 and ABCA1/ABCG1 proteins.	Enhance macrophage autophagy, promote cholesterol outflow and inhibit the activation of inflammasomes.	Diao (2021)
​	Dipsacoside B	ApoE^−/−^ mice; gavage for 8 weeks; 10 and 100 mg/kg/d; atorvastatin (10 mg/kg/d)	RAW264.7 cells; 50 h; 10, 30 and 100 μM; CQ	Increase the levels of LC3II/I ratio.	Improve lipid metabolism, induce autophagy of foam cells and inhibit lipid accumulation.	Quan et al. (2024)
Alkaloids	Berberine	SD rats; gavage for 4 weeks; 150 mg/kg/d; NC	J774A.1 cells; 24 h; 6.25, 12.5, 25 and 50 μM; CQ	1) Inhibit the AMPK/mTOR signaling pathway; 2) Increase the LC3II/I ratio and reduce SQSTM1/p62 protein expression.	Enhance macrophage autophagy, and inhibit inflammatory reaction and foam cell formation.	Fan et al. (2015), Ke et al. (2020)
​	​	ApoE^−/−^ mice; gavage for 12 weeks; 78,117 and 156 mg/kg/d; ATO + PIO (4.6 mg/kg/d)	peritoneal macrophages; 0–12 h; 50, 100, 150 and 200 μM; 3-MA	1) Regulate the PI3K/AKT/mTOR and SIRT1/TFEB signaling pathway; 2) Increase the LC3II/I ratio and Beclin1 expression; 3) Promote the degradation of SQSTM1/p62 protein.	1) Promote macrophage autophagy and suppress apoptosis; 2) Reduce the levels of blood lipids.	Song and Chen (2021), Zheng et al. (2021)
Terpenoids	Tanshinone IIA	ApoE^−/−^ mice; intraperitoneal injection for 12 weeks; 10 mg/kg/d; NC	RAW264.7 cells; 8, 16 and 24 h; 10 μg/μL; NC	1) Regulate the miR-375/KLF4 signaling pathway; 2) Increase the level of LC3II and Beclin1.	Reduce the levels of blood lipids, enhance macrophage autophagy and polarization, and inhibit the release of inflammatory factors.	Chen W. et al. (2019)
​	Artemisinin	ApoE^−/−^ mice; gavage for 8 weeks; 50 and 100 mg/kg/d; NC	RAW264.7 cells and BMDMs; 24 h; 100 μM; Compound C	1) Regulate the AMPK/mTOR/ULK1 pathway; 2) Increase the ratio of LC3II/I and decrease the level of p62 protein.	1) Enhance macrophage autophagy and inhibit foam cell formation; 2) Inhibit inflammatory response.	Cao Q. et al. (2020)
​	Ursolic acid	LDLR^−/−^ mice; injected intraperitoneally for 11 weeks; 50 mg/kg/d; NC	RAW264.7 cells and peritoneal macrophages; 6–24 h; 100 μM; NC	1) Increase the expression of Atg5 and Atg16l1; 2) Increase the ratio of LC3II/I.	Enhance macrophage lipophagy, promote cholesterol outflow, and inhibit the secretion of inflammatory factors.	Leng et al. (2016)
​	Ginkgolide C	ApoE^−/−^ mice; tail vein injection for 7 days; 12, 24 and 48 mg/kg/d; atorvastatin (2.5 mg/kg/d)	RAW264.7 cells; 24 h; 100 μM; NC	1) Promote autophagy mediated by molecular chaperone; 2) Increase the expression of LAMP-2A.	Improve blood lipids, enhance macrophage autophagy and inhibit the release of inflammatory factors.	Zhang (2025)
​	Gastrodin	No	RAW264.7 cells; 24 h; 100 μM; Dorsomorphin dihydrochloride	1) Regulate the AMPK-FoxO1-TFEB signaling pathway; 2) Increase the expression of LC3II, LAMP1, Beclin1, and ABCA1; 3) Suppress the expression of CD36 and p62.	Induce lysosomal biogenesis, enhance autophagic activity, promote cholesterol efflux, and inhibit lipid accumulation and inflammatory responses.	Tao J. et al. (2021)
Other Compounds	Chrysophanol	No	RAW264.7 cells; 24 h; 10 and 15 μM; NC	1) Inhibit the NF-κB/HMGB1-PI3K/AKT/mTOR signaling pathway; 2) Increase the expression of the LC3II/I ratio, ATG5, Beclin1, ABCA1 and ABCG1; 3) Inhibit the expression of CD36, SR-A1 and p62.	Enhance macrophage autophagy, promote cholesterol excretion, inhibit inflammation and lipid accumulation.	Wu et al. (2024)
​	*Laminaria japonica* polysaccharide	LDLR^−/−^ mice; gavage for 16 weeks; 50 and 100 mg/kg/d; simvastatin (5 mg/kg/d)	RAW264.7 cells; 24 h; 5 and 25 μg/mL; 3-MA	1) Activate the SIRT1/FoxO1 signaling pathway; 2) Increase the LC3II/I ratio and reduce p62 protein levels.	Enhance macrophage autophagy, promote macrophage polarization from M1 to M2 type, and reduce foam cell formation.	Li et al. (2022)
​	Polydatin	ApoE^−/−^ mice; gavage for 8 weeks; 50, 100 and 200 mg/kg/d; simvastatin (5 mg/kg/d)	No	1) Inhibit the NLRP3/mTOR pathway; 2) Upregulate the LC3II/I ratio and reduce p62 levels.	Regulate blood lipids, enhance autophagy, and suppress inflammation and pyroptosis.	Zhang X. et al. (2023)
​	Salvianolic acid B	No	RAW264.7 cells; 24 h; 100, 150, and 200 μM; NC	1) Inhibit the AKT/mTOR signaling pathway; 2) Increase LC3II and Beclin1 expression while downregulating p62 expression.	Improve macrophage autophagy function, reduce apoptosis and inflammatory cytokine release.	Sun et al. (2021)

**TABLE 2:** Mechanisms of TCM plants in regulating autophagy/lipophagy against atherosclerosis.

Botanical drugs	Major metabolites	*In vivo* (model; duration; dosage; control)	*In vitro* (model; duration; dosage; control)	Autophagy/lipophagy signaling pathways	Anti-AS mechanisms	References
*Trichosanthes kirilowii* Maxim. [Cucurbitaceae; Trichosanthis fructus] and *Allium macrostemon* Bunge [Amaryllidaceae; Allii macrostemonis bulbus]	albiflorin, apigenin, luteolin, kaempferol, 7,8-dihydroxyflavone, hesperetin	ApoE^−/−^ mice; 3, gavage for 4 weeks; 6 and 12 mg/kg/d; simvastatin (5 mg/kg/d)	RAW264.7 cells; 24 h; 0.5, 1, and 2 mg/mL; NC	1) Inhibit the P2RY12/PI3K signaling pathway; 2) Increase LC3II expression while downregulating p62 and Plin2 protein expression.	Enhance lipid phagocytosis, promote LD degradation, and suppress foam cell formation and inflammatory responses.	Bao et al. (2024), Bao et al. (2023)
*Salvia miltiorrhiza* Bunge [Lamiaceae; Salviae miltiorrhizae radix et rhizoma]	Absent profiling	ApoE^−/−^ mice; intraperitoneal injection for 8 weeks; 50 mg/kg/d; NC	RAW264.7 cells and HUVECs; 48 h; 30 μg/mL; NC	Promote the conversion of LC3I to LC3II and degradation of p62.	Enhance autophagy and lysosomal activity, and reduce foam cell formation.	Ko et al. (2020)
*Ganoderma lucidum* spore powder	Ganoderic acid A, Ganoderic acid C6, Ganoderic acid G, Ganodermanontriol	LDLR^−/−^ mice; mixed with HFD for 12 or 36 weeks; 1,400 mg/kg/d; atorvastatin (4 mg/kg/d)	RAW264.7 cells and HASMCs; 24 h; 10 μM; NC	Increase the expression of ABCA1 and ABCG1.	Promote cholesterol efflux, inhibit lipid deposition, increase collagen content, suppress inflammation, oxidative stress, and vascular calcification.	Zheng et al. (2023)

**TABLE 3:** Mechanisms of Chinese herbal formulas in regulating autophagy/lipophagy against atherosclerosis.

Chinese herbal formulas	Ingredients	*In vivo* (model; dosage; duration; control)	*In vitro* (model; dosage; duration; control)	Autophagy/lipophagy signaling pathways	Anti-AS mechanisms	References
Huanglian Jiedu Decoction	*Coptis chinensis* Franch. [Ranunculaceae; Coptidis rhizoma], *Scutellaria baicalensis* Georgi [Lamiaceae; Scutellariae radix], *Phellodendron chinense* C.K.Schneid. [Rutaceae; Phellodendri chinensis cortex], *Gardenia jasminoides* J.Ellis [Rubiaceae; Gardeniae fructus]	ApoE^−/−^ mice; 2.3, 4.6, and 9.2 g/kg/d; gavage for 8 weeks; atorvastatin (10 mg/kg/d) and clopidogrel hydrogen sulfate (20 mg/kg/d)	VSMCs and A7r5 cells; 200, 400, and 800 μg/mL; 24 h; NC	1) Inhibit the P2RY12/PI3K/AKT pathway; 2) Increase LC3II levels and decrease p62 levels.	Enhance lipophagocytosis, and inhibit lipid accumulation, apoptosis, and oxidative stress.	Lin et al., (2024a)
Zhishi Xiebai Guizhi Decoction	*Citrus aurantium* L. [Rutaceae; Aurantii fructus immaturus], *Trichosanthes kirilowii* Maxim. [Cucurbitaceae; Trichosanthis fructus], *Allium macrostemon* Bunge [Amaryllidaceae; Allii macrostemonis bulbus], *Magnolia officinalis* Rehder and E.H.Wilson [Magnoliaceae; Magnoliae officinalis cortex], Cinnamomum cassia (L.) J.Presl [Lauraceae; Cinnamomi ramulus]	ApoE^−/−^ mice; 2.0475, 4.095, and 8.19 g/kg/d; gavage for 8 weeks; simvastatin (2.60 mg/kg/d)	No	1) Modulate the AGE/RAGE signaling pathway; 2) Increase Beclin1 and LC3 expression.	Promote autophagy while inhibiting apoptosis and inflammation.	Zhang Z. et al. (2024)
Taoren Honghua Decoction	*Prunus persica* (L.) Batsch [Rosaceae; Persicae semen], *Carthamus tinctorius* L. [Asteraceae; Carthami flos], *Salvia miltiorrhiza* Bunge [Lamiaceae; Salviae miltiorrhizae radix et rhizoma], *Corydalis yanhusuo* (Y.H.Chou & Chun C.Hsu) W.T.Wang ex Z.Y.Su and C.Y.Wu [Papaveraceae; Corydalis rhizoma], *Boswellia sacra* Flück. [Burseraceae; Olibanum], *Citrus reticulata* Blanco [Rutaceae; Citri reticulatae pericarpium viride], *Paeonia lactiflora* Pall. [Paeoniaceae; Paeoniae radix rubra], [Paeoniaceae; Paeoniae radix alba], *Ligusticum chuanxiong* Hort. [Apiaceae; Chuanxiong rhizoma], *Angelica sinensis* (Oliv.) Diels [Apiaceae; Angelicae sinensis radix]	ApoE^−/−^ mice; 5.5 mg/kg/d; gavage for 12 weeks; atorvastatin (5 mg/kg/d)	RAW264.7 cells; 25, 50, and 100 μg/mL; 24 h; 3-MA	1) Inhibit the PI3K/AKT signaling pathway; 2) Increase expression of LC3II, ABCA1, and ABCG1 while downregulating p62 levels.	Enhance macrophage autophagy, promote cholesterol efflux and reduce inflammatory responses.	Chang et al. (2025)
Tiaozhi Tongmai Granules	*Astragalus mongholicus* Bunge [Fabaceae; Astragali radix], *Angelica sinensis* (Oliv.) Diels [Apiaceae; Angelicae sinensis radix], *Crataegus pinnatifida* Bunge [Rosaceae; Crataegi fructus], *Alisma orientale* (Sam.) Juz. [Alismataceae; Alismatis rhizoma], *Cassia obtusifolia* L. [Fabaceae; Cassiae semen], *Pheretima aspergillum* (E.Perrier) [Megascolecidae; Pheretima], *Panax notoginseng* (Burkill) F.H.Chen [Araliaceae; Notoginseng radix et rhizoma] (Sanqi)	ApoE^−/−^ mice; 16.5 g/kg/d; intragastric injection for 16 weeks; atorvastatin (5.1 mg/kg/d)	No	Increase LC3II and LAMP1 protein expression, and promote the fusion of autophagosomes, lysosomes, and LDs.	Enhance macrophage lipophagocytosis and inhibit plaque formation.	Zheng et al. (2015)
Sanjie Tongmai Formula	*Salvia miltiorrhiza* Bunge [Lamiaceae; Salviae miltiorrhizae radix et rhizoma], *Whitmania pigra* Whitman [Hirudinidae; Hirudo], *Panax notoginseng* (Burkill) F.H.Chen [Araliaceae; Notoginseng radix et rhizoma], *Fritillaria thunbergii* Miq. [Liliaceae; Fritillariae bulbus], *Artemisia capillaris* Thunb. [Asteraceae; Artemisiae scopariae herba], *Poria cocos* (Schw.) Wolf [Polyporaceae; Poria], *Paeonia lactiflora* Pall. [Paeoniaceae; Paeoniae radix alba], *Alisma orientale* (Sam.) Juz. [Alismataceae; Alismatis rhizoma], *Santalum album* L. [Santalaceae; Santali albi lignum], *Bombyx mori L. [Bombycidae; Bombyx batryticatus]*, *Cryptotympana atrata* (Fabricius) [Cicadidae; Cicadae periostracum], *Citrus aurantium* L. [Rutaceae; Aurantii fructus], *Citrus reticulata* Blanco [Rutaceae; Citri reticulatae pericarpium], *Bambusa tuldoides* Munro [Poaceae; Bambusae caulis in taeniam]	C57BL/6J mice; 40.24 g/kg/d; gavage for 4 weeks; atorvastatin (3 mg/kg)	No	1) Inhibit the PI3K/AKT/mTOR pathway; 2) Increase LC3II/I and Beclin1 levels while decreasing p62 levels.	Improve lipid profiles, enhance macrophage autophagy, promote macrophage polarization toward the M2 phenotype, and suppress inflammatory responses.	Li et al. (2023)
Bushen Jiangzhi Formula	*Cuscuta chinensis* Lam. [Convolvulaceae; Cuscutae semen], *Astragalus complanatus* Bunge [Fabaceae; Astragali complanati semen], *Rubus chingii Hu* [Rosaceae; Rubi fructus], *Ligustrum lucidum* W.T.Aiton [Oleaceae; Ligustri lucidi fructus], *Lycium barbarum* L. [Solanaceae; Lycii fructus], *Glycine* max (L.) Merr. [Fabaceae; Sojae semen nigrum]	ApoE^−/−^ mice; 12.33 g/kg/d; gavage for 12 weeks; atorvastatin (2.05 mg/kg/d)	No	Increase LC3II/I and Beclin1 levels while decreasing p62 levels.	Regulate blood lipids, enhance autophagy, reduce lipid accumulation, increase collagen fiber content, and suppress inflammatory responses.	Cao et al. (2020)
Bushen Kangshuai tablets	*Poria cocos* (Schw.) Wolf [Polyporaceae; Poria], *Ligusticum chuanxiong* Hort. [Apiaceae; Chuanxiong rhizoma], *Citrus reticulata* Blanco [Rutaceae; Citri reticulatae pericarpium viride], *Cinnamomum cassia* (L.) J.Presl [Lauraceae; Cinnamomi cortex], *Codonopsis pilosula* (Franch.) Nannf. [Campanulaceae; Codonopsis radix], *Mauremys reevesii* (Gray) [Geoemydidae; Testudinis carapax et plastrum], *Acorus tatarinowii* Schott [Acoraceae; Acori tatarinowii rhizoma], *Salvia miltiorrhiza* Bunge [Lamiaceae; Salviae miltiorrhizae radix et rhizoma], *Sargassum fusiforme* (Harv.) Setch. [Sargassaceae; Sargassum], *Laminaria japonica* Aresch. [Laminariaceae; Laminariae thallus], *Cuscuta chinensis* Lam. [Convolvulaceae; Cuscutae semen], *Prunella vulgaris* L. [Lamiaceae; Prunellae spica], *Polygonum multiflorum* Thunb. [Polygonaceae; Polygoni multiflori radix praeparata], *Taxillus chinensis* (DC.) Danser [Loranthaceae; Taxilli herba], *Eucommia ulmoides* Oliv. [Eucommiaceae; Eucommiae cortex]	No	RAW264.7 cells; 10% drug-containing serum; 24 h; Rap	1) Inhibit the PI3K/AKT/mTOR pathway; 2) Increase LC3II/I levels while decreasing p62 levels.	Enhance macrophage autophagy, promote the polarization of macrophages from M1 to M2 type, and suppress inflammatory cytokine secretion.	Xie et al. (2020)
Huayu Qutan Formula	*Codonopsis pilosula* (Franch.) Nannf. [Campanulaceae; Codonopsis radix], *Astragalus mongholicus* Bunge [Fabaceae; Astragali radix], *Gynostemma pentaphyllum* (Thunb.) Makino [Cucurbitaceae; Herba Gynostemmatis Pentaphylli], *Salvia miltiorrhiza* Bunge [Lamiaceae; Salviae miltiorrhizae radix et rhizoma], *Poria cocos* (Schw.) Wolf [Polyporaceae; Poria], *Pinellia ternata* (Thunb.) Makino [Araceae; Pinelliae rhizoma], *Acorus tatarinowii* Schott [Acoraceae; Acori tatarinowii rhizoma], *Ligusticum chuanxiong* Hort. [Apiaceae; Chuanxiong rhizoma], *Curcuma wenyujin* Y.H.Chen and C.Ling [Zingiberaceae; Curcumae radix]	ApoE^−/−^ mice; 10.27, 20.54, and 41.08 g/kg/d; gavage for 8 weeks; simvastatin (10 mg/kg/d)	RAW264.7 cells; 10% drug-containing serum; 24 h; CQ and Rap	1) Modulate the mTORC1/TFEB signaling pathway; 2) Increase the expression of LC3II, Beclin1, LAMP1, SCARB1, ABCA1, and ABCG1 while reducing p62 levels.	Enhance macrophage lipophagy, regulate blood lipids, promote cholesterol efflux, and inhibit foam cell formation.	Li et al. (2025), Li et al. (2024)

**TABLE 4:** Mechanisms of CCPPs in regulating autophagy/lipophagy against atherosclerosis.

CCPPs	Ingredients	*In vivo* (model; dosage; duration; control)	*In vitro* (model; dosage; duration; control)	Autophagy/Lipophagy signaling pathways	Anti-AS mechanisms	References
Danlou tablets	*Trichosanthes kirilowii* Maxim. [Cucurbitaceae; Trichosanthis pericarpium], *Allium macrostemon* Bunge [Amaryllidaceae; Allii macrostemonis bulbus], *Pueraria montana* var. lobata (Willd.) Maesen and S.M.Almeida ex Sanjappa & Predeep [Fabaceae; Puerariae lobatae radix], *Ligusticum chuanxiong* Hort. [Apiaceae; Chuanxiong rhizoma], *Salvia miltiorrhiza* Bunge [Lamiaceae; Salviae miltiorrhizae radix et rhizoma], *Paeonia lactiflora* Pall. [Paeoniaceae; Paeoniae radix rubra], *Alisma orientale* (Sam.) Juz. [Alismataceae; Alismatis rhizoma], *Astragalus mongholicus Bunge [Fabaceae; Astragali radix]*, *Drynaria fortunei* (Kunze) J.Sm. [Polypodiaceae; Drynariae rhizoma], *Curcuma wenyujin* Y.H.Chen and C.Ling [Zingiberaceae; Curcumae radix]	ApoE^−/−^ mice; 120, 240, and 480 μg/kg/d; gavage for 16 weeks; atorvastatin (2 mg/kg)	RAW264.7 cells and BMDMs; 7.5, 75, and 750 μg/mL; 24 h; Torin 1	1) Inhibit the PI3K/AKT/mTOR pathway; 2) Increase LC3II/I levels and decrease p62 levels.	Enhance macrophage autophagy, regulate blood lipids, reduce lipid accumulation, and suppress inflammatory responses.	Liu C. et al. (2021)
Sanwei Tanxiang capsule	*Santalum album* L. [Santalaceae; Santali albi lignum], *Choerospondias axillaris* (Roxb.) B.L.Burtt and A.W.Hill [Anacardiaceae; Choerospondiatis fructus], *Myristica fragrans* Houtt. [Myristicaceae; Myristicae semen]	ApoE^−/−^ mice; 1.75 and 3.5 g/kg/d; 5 or 8 weeks; NC	RAW264.7 cells; 40 μg/mL (esculetin and morin); 24 h; CQ	Enhance macrophage lysosomal activity.	Enhance macrophage cholesterol efflux, increase postprandial HDL-C levels and improve plaque stability.	Ye et al. (2023)
Shenyuan Dan	*Salvia miltiorrhiza* Bunge [Lamiaceae; Salviae miltiorrhizae radix et rhizoma], *Astragalus mongholicus* Bunge [Fabaceae; Astragali radix], *Codonopsis pilosula* (Franch.) Nannf. [Campanulaceae; Codonopsis radix], *Scrophularia ningpoensis* Hemsl. [Scrophulariaceae; Scrophulariae radix], *Whitmania pigra* Whitman [Hirudinidae; Hirudo], *Pheretima aspergillum* (E.Perrier) [Megascolecidae; Pheretima], *Eupolyphaga sinensis* Walker [Corydiidae; Eupolyphaga steleophaga], *Corydalis yanhusuo* (Y.H.Chou & Chun C.Hsu) W.T.Wang ex Z.Y.Su and C.Y.Wu [Papaveraceae; Corydalis rhizoma]	ApoE^−/−^ mice; 40, 80, and 160 mg/kg/d; gavage for 6 weeks; atorvastatin (3.34 mg/kg/d)	RAW264.7 cells; 1.5625, 3.125, and 6.25 mg/mL; 24 h; NC	1) Inhibit the PI3K/AKT/mTORC1 signaling pathway; 2) Increase expression of LC3II, Beclin1, and Atg13.	Regulate blood lipids, enhance macrophage autophagy activity, reduce cholesterol accumulation and foam cell formation.	Zhou et al. (2019)
Tianxiang Dan	*Ziziphora bungeana* Juz [Lamiaceae; Ziziphorae herba], *Rhodiola crenulata* (Hook.f. and Thomson) H.Ohba [Crassulaceae; Rhodiolae crenulatae radix et rhizoma], *Salvia miltiorrhiza* Bunge [Lamiaceae; Salviae miltiorrhizae radix et rhizoma], *Dalbergia odorifera* T.C.Chen [Fabaceae; Dalbergiae odoriferae lignum]	ApoE^−/−^ mice; 2.73 g/kg/d; gavage for 8 weeks; atorvastatin (6.06 mg/kg/d)	THP-1 cells; 75 μg/mL; 24 h; LY294002	1) Inhibit the PI3K/AKT/mTORC signaling pathway; 2) Increase expression of TFEB, LC3II/I, ULK1, and ABCA1.	Regulate blood lipids, enhance lipophagy, promote cholesterol efflux, reduce foam cell formation and arterial lipid deposition.	(Zhang Y. et al. (2025)
Tongxinluo	*Panax ginseng* C.A.Mey. [Araliaceae; Ginseng radix et rhizoma], *Whitmania pigra* Whitman [Hirudinidae; Hirudo], *Buthus martensii* Karsch [Buthidae; Scorpio], *Paeonia lactiflora* Pall. [Paeoniaceae; Paeoniae radix rubra], *Cryptotympana atrata* (Fabricius) [Cicadidae; Cicadae periostracum], *Eupolyphaga sinensis* Walker [Corydiidae; Eupolyphaga steleophaga], *Scolopendra subspinipes mutilans* L.Koch [Scolopendridae; Scolopendra], *Santalum album* L. [Santalaceae; Santali albi lignum], *Dalbergia odorifera* T.C.Chen [Fabaceae; Dalbergiae odoriferae lignum], *Boswellia sacra* Flück. [Burseraceae; Olibanum], *Ziziphus jujuba* Mill. var. spinosa (Bunge) Hu ex H.F.Chow [Rhamnaceae; Ziziphi spinosae semen], *Borneolum Syntheticum* (Bingpian)	ApoE^−/−^ mice; 0.38, 0.75, and 1.5 g/kg/d; gavage for 8 weeks; NC	THP-1 cells; 100, 200, and 500 mg/L; 36 h; NC	1) Regulate the HDAC/Beclin1 pathway; 2) Increase LC3II, the LC3II/I ratio, and Beclin1 protein expression, promoting the fusion of autophagosomes with lysosomes; 3) Increase ABCA1/ABCG1 expression and downregulate LOX-1 expression.	Enhance macrophage lipophagy, promote LD degradation and cholesterol efflux, inhibit apoptosis, and improve plaque stability.	Chen et al. (2018), Chen et al. (2021)

## 3 Mechanism of macrophage lipophagy

### 3.1 Classification of lipophagy

LDs are dynamic organelles rather than inert lipid storage depots. They interact with lysosomes, endoplasmic reticulum, mitochondria, and other cellular structures, which play a crucial role in maintaining lipid homeostasis and signal transduction (Olzmann and Carvalho, 2019). The core characteristic of lipophagy is the sequestration of LDs by autophagosomes and then transportation to lysosomes for degradation. This process releases metabolites such as free fatty acids and triglycerides to maintain cellular energy balance and lipid homeostasis (Liu and Czaja, 2013; Singh et al., 2009). Based on the pathway by which substrates enter the lysosome, lipophagy is classified into three types: macrolipophagy, microlipophagy, and lipophagy mediated by chaperone-mediated autophagy (CMA) (Laval and Ouimet, 2023; Zhao R. et al., 2026) (Figure 1).

**FIGURE 1:**
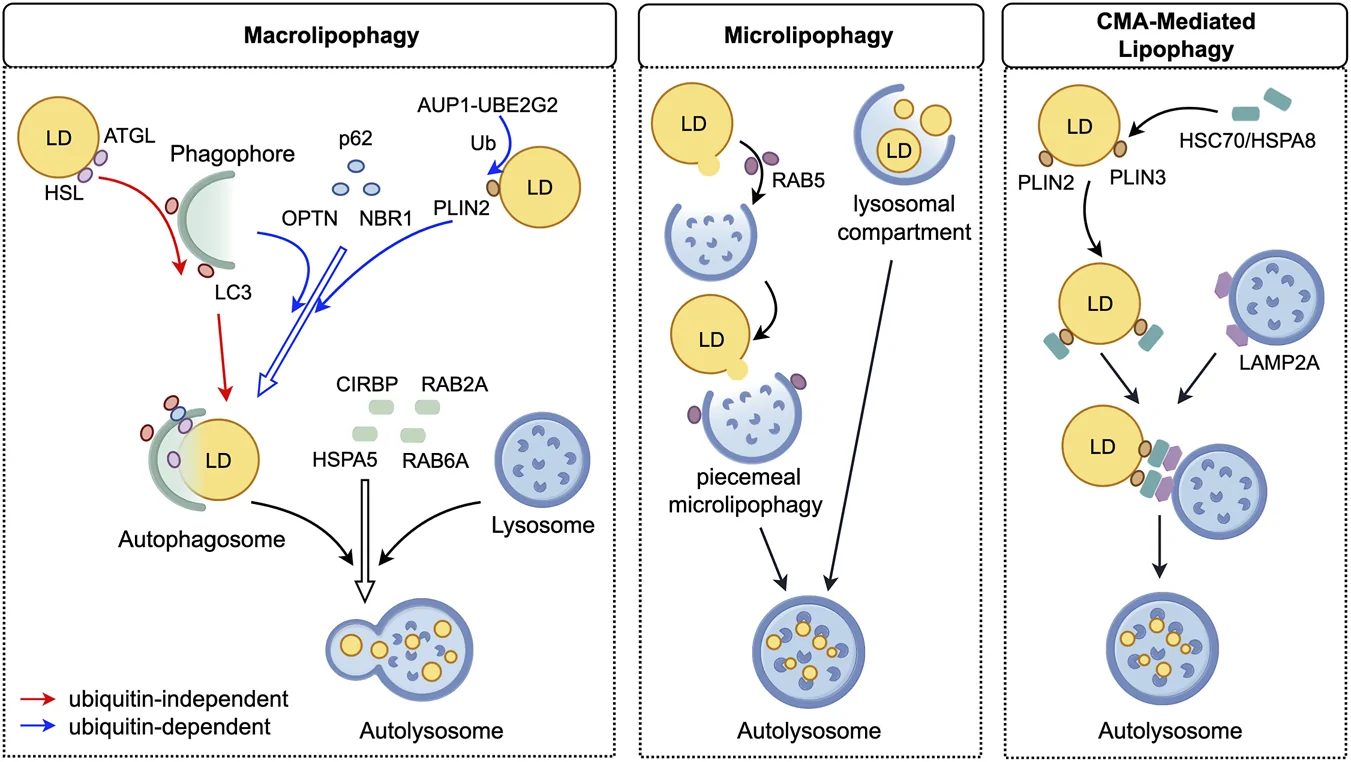
Three types of lipophagy pathways. Macrolipophagy forms autophagosomes around lipid droplets (LDs) via ubiquitin-dependent or -independent mechanisms. Microlipophagy directly engulfs LDs by lysosomal membrane invagination, regulated by Rab5 in animal cells. Chaperone-mediated autophagy (CMA) degrades perilipin 2 or 3 (PLIN2/PLIN3) via heat-shock protein 70 (HSC70) recognition and lysosomal-associated membrane protein type 2A (LAMP2A)-mediated translocation.

#### 3.1.1 Macrolipophagy

Macrolipophagy is a prevalent form of lipophagy in mammals (Rahman et al., 2021). Similar to macroautophagy, it initiates with the creation of a double membrane structure in cytoplasm, which expands and sequesters LDs or LD fragments to form a mature autophagosome. Subsequently, the autophagosome merges with the lysosome, delivering its encapsulated cytoplasmic contents into the lysosomal lumen for degradation (Yamamoto and Matsui, 2024). In the process of macrolipophagy, LDs can be selectively identified via either ubiquitin-dependent or ubiquitin-independent mechanisms.

In the ubiquitin-dependent pathway, autophagosomes are recruited to LDs via selective autophagy receptors (SARs), including SQSTM1/p62, OPTN, and NBR1. These receptors bind to the ubiquitin tags on the LD surface proteins and concurrently interact with LC3 via LC3-interacting regions (LIRs). This process is facilitated by ancient ubiquitous protein 1 (AUP1), which recruits the ubiquitin-conjugating enzyme E2 G2 (UBE2G2) to catalyze ubiquitination of perilipin 2 (PLIN2) or other substrates (Shroff and Nazarko, 2023; Yamamoto and Matsui, 2024). Together with lipophagy factors such as HSPA5, RAB2A, RAB6A, and CIRBP, these components assemble into a lipophagic complex that engages LC3 and drives autophagosome biogenesis. The resulting LD-containing autophagosome merges with lysosomes, where lysosomal acid lipase (LAL) catalyzes lipid breakdown. This pathway contributes to lipophagy enhancement and promotes cholesterol efflux from macrophage foam cells (Robichaud et al., 2021). In the ubiquitin-independent pathway, adipose triglyceride lipase (ATGL) and hormone-sensitive lipase (HSL), which are located on the phospholipid monolayer of LDs, directly interact with LC3 to induce the local formation of phagophores around the LDs (Filali-Mouncef et al., 2022). However, the interplay between ubiquitin-dependent and ubiquitin-independent macrolipophagy remains unclear.

#### 3.1.2 Microlipophagy

LDs are targeted to lysosomes via macrolipophagy or microlipophagy, which is coordinated by ubiquitination, lipophagy selectivity factors, and autophagy receptors. While macrolipophagy has long been regarded as the primary lipophagic pathway and thus extensively studied, microlipophagy has received less attention due to its initial characterization in lower organisms such as yeast (Kumar et al., 2025). In fact, microlipophagy also exists in mammals and can be triggered by nutrient deprivation or endoplasmic reticulum stress. It not only supplies fatty acids to sustain cell growth but also aids in maintaining endoplasmic reticulum homeostasis by removing misfolded proteins (Rahman et al., 2021; Garcia et al., 2021).

In *Saccharomyces cerevisiae*, stimulation by oleic acid or starvation enhances the activity of the autophagy-related 15 (ATG15), which promotes the vacuolar engulfment and LDs degradation through the microlipophagy process (van Zutphen et al., 2014). Yeast microlipophagy relies on sterol-enriched vacuolar membrane microdomains, which are formed with the involvement of Ncr1, Npc2, and ATG proteins, to directly engulf LDs (Filali-Mouncef et al., 2022; Kim et al., 2026). In animal cells, Rab5 plays an important role in microlipophagy by directing LDs to lysosomes (Kuchitsu and Taguchi, 2024). During microlipophagy, the lysosomal membrane invaginates, protrudes, or forms tubular structures to directly encapsulate adjacent cytoplasmic constituents, generating single-membrane vesicles known as microautophagic bodies. These vesicles and their contents are subsequently exposed to hydrolytic enzymes for breakdown (Yamamoto and Matsui, 2024; Kuchitsu and Taguchi, 2024). LDs can be directly engulfed and degraded by lysosomes through a piecemeal-type microlipophagy process. Alternatively, they can be enclosed as clusters within lysosomal compartments for degradation (Robichaud et al., 2021).

However, direct evidence for microlipophagy in macrophage foam cells remains limited compared with macrolipophagy and CMA. Most mammalian microlipophagy studies have been conducted in hepatocytes and other cell types, and its specific contribution to LD turnover in atherosclerotic macrophages requires further investigation (Schulze et al., 2020).

#### 3.1.3 CMA-mediated lipophagy

CMA, a highly selective form of autophagy primarily occurring in mammalian cells, regulates lipophagy by degrading proteins on the surface of LDs (Mastoridou et al., 2023). In both human and mouse AS models, the function of CMA is impaired (Madrigal-Matute et al., 2022). This dysfunction has been shown to exacerbate vascular inflammation and atherosclerotic lesions through activation of the NLRP3 inflammasome (Qiao et al., 2021).

The substrates of CMA contain a specific pentapeptide motif in their amino acid sequence, which is recognized and bound by the cytosolic constitutive heat-shock protein 70 (HSC70, also called HSPA8) and its co-chaperones. HSC70 functions as an ATP-dependent molecular chaperone, assisting in substrate protein folding, assembly, transport, and degradation (Stricher et al., 2013; Silva et al., 2021). The resulting substrate-chaperone complex is then targeted to the lysosomal membrane, where it binds to the lysosomal-associated membrane protein type 2A (LAMP2A), a single-pass transmembrane protein localized on the lysosomal membrane. Subsequent multimerization of the complex induces the formation of a protein-translocation channel, enabling the unfolded substrate to cross the membrane into the lysosomal lumen for degradation (Yamamoto and Matsui, 2024; Kirchner et al., 2019).

Perilipins (PLINs) are phosphorylated proteins on LDs. By shielding the internal triacylglycerol core from the surrounding hydrophilic cytoplasmic environment, they serve as one of the lipid-anchored protein substrates for CMA (Kaushik and Cuervo, 2016). Studies indicate that clearance of PLIN proteins via CMA is a prerequisite for initiating lipolysis through cytosolic lipases or the lipophagy pathway. Impaired CMA function leads to LDs accumulation both *in vitro* and *in vivo* (Kaushik and Cuervo, 2015). Specifically, PLIN2 initially binds to the chaperone HSC70 and subsequently undergoes AMP-activated protein kinase (AMPK) dependent phosphorylation, thereby activating the CMA degradation pathway. This process facilitates the recruitment of cytosolic lipases and autophagy-related effector proteins to LDs, consequently inducing lipophagy (Kaushik and Cuervo, 2016). Notably, CMA is also associated with the process of macrolipophagy. After the degradation of PLIN2 and PLIN3 via CMA, the levels of cytosolic ATGL and macroautophagy-related proteins increase and accumulate on LDs, which can further promote macrolipophagy progress (Kaushik and Cuervo, 2015).

### 3.2 Differences between lipophagy and cytosolic lipolysis

In macrophages, LD degradation is accomplished through two principal pathways: cytosolic lipolysis and lipophagy. Cytosolic lipolysis is mediated by neutral lipases, including ATGL, HSL, and monoacylglycerol lipase (MGL), which act sequentially at the LD surface to hydrolyze triglycerides into free fatty acids and glycerol (Getiye et al., 2022). In contrast, lipophagy delivers intact LDs to lysosomes, where LAL and other hydrolases degrade lipids in an acidic environment (Singh et al., 2009). These two pathways are functionally coupled. CMA-mediated degradation of PLIN2 and PLIN3 on the LD surface is a prerequisite for the recruitment of cytosolic lipases and the subsequent initiation of both cytosolic lipolysis and macrolipophagy (Kaushik and Cuervo, 2015; Kaushik and Cuervo, 2016). Conversely, lipophagy-derived free fatty acids can feedback-regulate lipase activity and LD biogenesis. In macrophage foam cells under chronic lipid overload, impaired lipophagy shifts lipid clearance toward cytosolic lipolysis, which may become insufficient, leading to LD accumulation and foam cell expansion (Getiye et al., 2022). The precise molecular crosstalk between these pathways in atherosclerotic macrophages remains an active area of investigation.

### 3.3 Signaling pathways of lipophagy regulation

Lipophagy plays a crucial role in both physiological and pathological conditions and is strongly associated with various metabolic disorders. Particularly during nutrient deprivation, it serves as an energy source by degrading LDs (Shin, 2020). Here we present several classical lipophagy-associated molecules and pathways (Figure 2).

**FIGURE 2:**
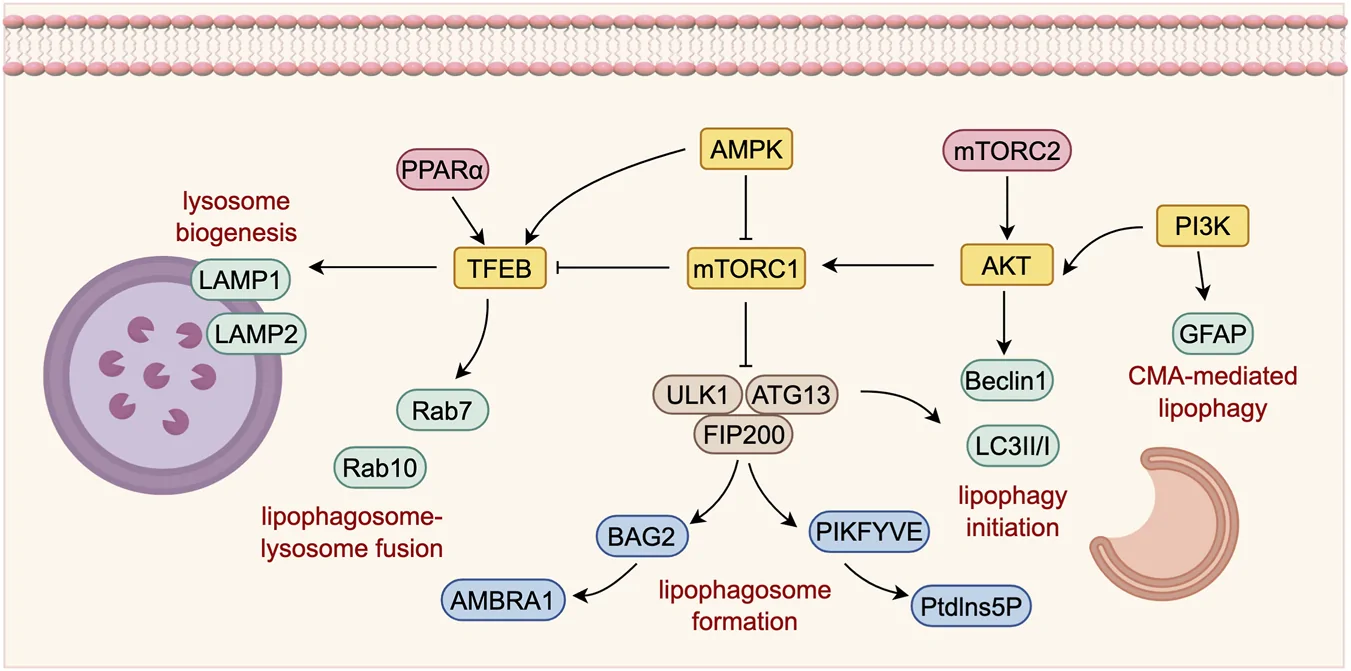
Signaling network regulating macrophage lipophagy. The AMPK energy-sensing axis activates the ULK1 complex to drive lipophagosome formation, while inhibiting mTORC1 and promoting TFEB-mediated lysosomal biogenesis. The PI3K/AKT growth factor axis suppresses autophagy via mTORC1 inhibition and direct Beclin1 phosphorylation at Ser295, and modulates GFAP-dependent CMA. The PPARα/TFEB axis upregulates lysosomal genes and Rab GTPases to enhance lysosomal biogenesis and lipophagosome–lysosome fusion.

#### 3.3.1 AMPK/ULK1 signaling pathway

AMPK is a serine/threonine kinase that functions as a cellular energy sensor. Upon activation under low energy conditions, it phosphorylates numerous substrates to restore energy homeostasis by regulating autophagy, lipid metabolism, and protein synthesis (Townsend and Steinberg, 2023). UNC-51-like autophagy-activating kinase 1 (ULK1), the mammalian homolog of autophagy related 1 (ATG1), is essential for autophagosome biogenesis. By forming a complex with ATG13 and FIP200, ULK1 phosphorylates autophagy proteins at specialized endoplasmic reticulum subdomains (Zou et al., 2022; Sankar et al., 2024). For example, ULK1 phosphorylates Bcl2-associated athanogene 2 (BAG2), which promotes the localization of the autophagy and Beclin 1 regulator 1 (AMBRA1) to the endoplasmic reticulum and positively regulates autophagy (Sankar et al., 2024). Furthermore, ULK1 phosphorylates phosphatidylinositol-3-phosphate 5-kinase (PIKFYVE), a kinase that regulates the synthesis of PI(3,5)P_2_, lysosomal function, and endocytic trafficking. This increases the formation of PI(5)P phospholipids and autophagosomes (Karabiyik and Rubinsztein, 2021). ULK1 also regulates autophagy through interactions with phosphatases. It can phosphorylate striatin (STRN), a regulatory subunit of protein phosphatase 2 (PPP2). Activated PPP2 subsequently removes inhibitory phosphorylation from ULK1 and promotes autophagy (Sankar et al., 2022). The activity of ULK1 is regulated by phosphorylation at multiple sites, which can exert opposing effects on autophagy (Kim et al., 2011). When AMPK phosphorylates ULK1 at Ser555, it increases the expression of autophagy markers LC3II/I and Beclin1, thus activating lipophagy (Gao et al., 2020; Fan et al., 2024). However, phosphorylation of ULK1 at Ser556 and Thr660 stabilizes the AMPK-ULK1 interaction, which inhibits the activation of ULK1 and vacuolar sorting protein 34 (VPS34), negatively regulating autophagy (Park et al., 2023).

CD36, a member of the scavenger receptor family, is a multifunctional membrane protein that promotes the uptake of long-chain fatty acids and triggers inflammatory responses, playing a significant role in AS pathogenesis (Park, 2014). CD36 functions as an upstream negative regulator of the AMPK/ULK1 pathway, which decreases LC3II expression and autophagosome formation while leading to p62 and lipid droplet accumulation. Conversely, CD36 deficiency promotes the phosphorylation of AMPKα (Thr172) and ULK1 (Ser555) to enhance lipophagic flux and alleviate lipid accumulation (Li et al., 2019).

Beyond its direct regulation of the ULK1 signaling pathway, AMPK can also influence lipophagy indirectly by modulating mammalian target of rapamycin (mTOR), a key regulatory protein that responds to anabolic growth signals while inhibiting catabolic processes and autophagy (Zhu et al., 2019). The mTOR complex 1 (mTORC1) phosphorylates ULK1 at Ser757, which disrupts the interaction between ULK1 and AMPK, thereby preventing AMPK-mediated activation of ULK1. However, AMPK promotes autophagy by inhibiting the activity of mTORC1 indirectly (Kim et al., 2011).

#### 3.3.2 PI3K/AKT signaling pathway

Phosphatidylinositol 3-kinase (PI3K) is a family of lipid kinases composed of catalytic and regulatory subunits. It phosphorylates intracellular inositol lipids to regulate signal transduction and intracellular vesicular trafficking, broadly modulating cell survival, growth, proliferation, and metabolism (Bilanges et al., 2019). Human cells express three classes of PI3K: Class I generates 3-phosphoinositide lipids to directly activate downstream signaling cascades, whereas Class II and Class III primarily regulate membrane transport within the endocytic pathway, participating in endosomal recycling and autophagy to exert indirect effects on cellular signaling (Safaroghli-Azar et al., 2023). Typically, PI3K can be activated by receptor-coupled tyrosine kinase activity, small Ras-related GTPases, or heterotrimeric G proteins (Bilanges et al., 2019; Fruman et al., 2017). Protein kinase B (AKT) serves as a central effector of the PI3K signaling pathway, regulating cell proliferation, metabolism, survival, and motility through phosphorylation at multiple sites (Zhang et al., 2026).

The PI3K/AKT pathway regulates autophagy via mTOR signaling. Upon activation, PI3K generates the lipid second messenger PIP_3_ and recruits AKT to the plasma membrane. This recruitment enables mTOR complex 2 (mTORC2) to phosphorylate AKT at Ser473, leading to its full activation (Wang S. et al., 2024; Glaviano et al., 2023). Under nutrient-rich conditions, activated AKT stimulates mTORC1 and then phosphorylates ULK1 at Ser757. This inhibits the assembly of the ULK1 complex and blocks AMPK-mediated activation of ULK1, effectively suppressing autophagy (Zhu et al., 2019; Wang S. et al., 2024). Conversely, nutrient deprivation reduces mTORC1 activity, which promotes autophagosome formation and induces autophagy (Seo et al., 2025). Furthermore, AKT can inhibit autophagy through the mTOR-independent mechanism, phosphorylating and inactivating Beclin1 at Ser295 (Wang et al., 2012). Alternatively, AKT directly phosphorylates ULK1 at Ser774, further suppressing the autophagic process (Bach et al., 2011). In addition to inhibiting macroautophagy, the PI3K/AKT pathway can suppress CMA by phosphorylating the glial fibrillary acidic protein (GFAP) on the lysosomal membrane (Huang and Wang, 2024).

#### 3.3.3 TFEB-mediated lysosomal-autophagic pathway

Transcription factor EB (TFEB) and transcription factor binding to IGHM enhancer 3 (TFE3) are master transcriptional regulators that govern autophagy and lysosomal activity. These factors participate in processes such as lysosome-autophagosome biogenesis, metabolic regulation, and cellular stress responses (Raben and Puertollano, 2016). TFEB can activate the macrophage autophagic-lysosomal system, which reduces the accumulation of ubiquitinated and SQSTM1/p62-rich protein aggregates, suppresses inflammatory responses and macrophage apoptosis, to alleviate AS (Evans et al., 2018). The nuclear localization and transcriptional activity of TFEB are regulated by multisite phosphorylation, which is governed by AMPK, mTORC1, and PPARα.

AMPK not only directly phosphorylates and activates TFEB/TFE3 but also suppresses mTORC1 activity to promote their nuclear translocation, leading to the activation of autophagy and lysosomal biogenesis (Paquette et al., 2021). When lysosomal damage occurs, the luminal glycans on LAMP1/LAMP2 proteins are exposed, recruiting AMPK to the lysosomal surface. Concurrently, the inhibition of the deubiquitinase USP9X causes increased ubiquitin clustering on the lysosomal membrane, which triggers autophagosome engulfment and AMPK activation (Jia et al., 2020). Activated AMPK enhances lysosome-associated autophagy by phosphorylating ULK1 to clear compromised organelles, while simultaneously promoting the biogenesis of new lysosomes via TFEB/TFE3-mediated transcription to maintain organellar homeostasis (Paquette et al., 2021; Steinberg and Hardie, 2023).

In macrophages, PPARα directly binds the TFEB promoter to upregulate its expression, recruiting the VPS34–Beclin1 complex, promoting autophagy, and suppressing AS (Tao H. et al., 2021). In hepatocytes, PPARα promotes the nuclear translocation of TFEB/TFE3 by enhancing Ca^2+^-mediated dephosphorylation. This process upregulates the expression of lysosomal membrane proteins LAMP1 and LAMP2A, as well as lipophagy-related genes such as Rab7a and Rab10. As a result, it induces lysosomal biogenesis and lipophagy to reduce cellular lipid accumulation (Yoo et al., 2021). The Rab GTPase family serves as a major regulator of intracellular vesicular trafficking and plays a critical role in lipophagy (Abba and Colombo, 2025; Shi et al., 2019). Generally, Rab7 facilitates the fusion of autophagosomes with lysosomes, participates in autophagic vesicle maturation, and promotes interactions between LDs and degradative organelles (Shi et al., 2019; Shin, 2020). Rab10 binds to the adaptor protein EHBP1 and the membrane-deforming ATPase EHD2 to promote autophagic membrane extension and LD degradation during lipophagy (Li et al., 2016).

#### 3.3.4 Other lipophagy-related pathways and molecules

The Hedgehog signaling pathway is a crucial signal transduction system that governs embryonic development, cell differentiation, and tissue regeneration. Importantly, it plays a key role in autophagy and lipid metabolism (VanDerMolen et al., 2025). Activation of Hedgehog signaling exacerbates lipid deposition and oxidative stress in foam cells. In contrast, Hedgehog pathway inhibition accelerates autophagosome maturation and cholesterol efflux in macrophage-derived foam cells, which reduces foam cell formation and attenuates early AS (Zhang Y. et al., 2023).

An increasing number of molecules have been identified to participate in macrophage lipophagy. Sirtuin 6 (SIRT6) is an NAD^+^-dependent histone deacetylase involved in the regulation of DNA damage repair and cellular senescence. It can attenuate foam cell formation and reduce AS risk by enhancing both autophagic flux and cholesterol efflux in macrophages (Lu et al., 2025). Mechanistically, SIRT6 recruits the chromatin remodeler sucrose non-fermenting protein 2 homologue (SNF2H) to downregulate Wnt1 expression. This event promotes LD degradation, stimulates macrophage lipophagy and mitigates AS (Wang et al., 2023). ATG14 is a core component of the class III PI3K complex I (PI3KC3-C1) that initiates autophagy. It facilitates the fusion of autophagosomes with lysosomes, enhances macrophage autophagy, and alleviates AS (Zhang et al., 2021). Moreover, ATG14 targets LDs by interacting with LC3/ATG8 and functions as an autophagy receptor to facilitate lipophagy (Yuan et al., 2024). Oxysterol-binding protein-related protein 8 (ORP8) is an endoplasmic reticulum-resident lipid transfer protein that mediates bidirectional cholesterol transport. Additionally, ORP8 acts as a lipophagy receptor by targeting LDs and participating in cellular lipid metabolism. It can be directly phosphorylated by AMPK and interact with LC3/GABARAP proteins, promoting the autophagic recognition and degradation of LDs (Pu et al., 2022). Programmed cell death 4 (PDCD4) has been shown to inhibit autophagy flux induced by ox-LDL in macrophages, subsequently promoting the transformation of macrophages into foam cells in an autophagy-dependent manner (Wang et al., 2016). Besides, customized siRNA arrays have identified a broader range of lipophagy regulators, including molecular chaperones (HSP90AA1, HSBP1, HSPA5, HSPH1), HMGB1/HMGB2, lysosomal proteins (NPC2, SCARB2), stress granule proteins (CIRBP, FUS), among others. These factors are closely associated with LD catabolism and macrophage lipophagy (Robichaud et al., 2021).

In summary, lipophagy selectively degrades LDs to maintain cellular energy homeostasis and lipid balance. Its regulatory mechanisms are complex and diverse, involving multiple signaling pathways and molecules (Figure 2). Elucidating the molecular mechanisms underlying lipophagy and its crosstalk with other pathogenic pathways in AS will contribute to the identification of novel therapeutic targets.

## 4 Macrophage lipophagy and atherosclerosis

### 4.1 Macrophage lipophagy inhibits foam cell formation

Foam cell formation is a key event in atherogenesis. It has been demonstrated in macrophage foam cells that, in addition to the classical neutral cholesteryl esters (CEs) hydrolase pathway, lipophagy delivers cytosolic lipids to lysosomes, where CEs are hydrolyzed by LAL to generate free cholesterol, which is then effluxed via the ABCA1 pathway to participate in reverse cholesterol transport (RCT) (Ouimet et al., 2011). During lipophagy, autophagosomes engulf triglycerides and cholesterol stored in LDs and deliver them to lysosomes for degradation. This process improves lipid metabolism, reduces lipid accumulation, decreases foam cell generation, and delays atherosclerotic progression (Li et al., 2026). However, impaired macrophage lipophagic flux disrupts cholesterol ester hydrolysis and diminishes cholesterol efflux, leading to increased foam cell burden and accelerated atherosclerotic plaque development (Jeong et al., 2017a).

Macrophage lipophagy counteracts foam cell formation by promoting cholesterol efflux, a key step in RCT for cholesterol homeostasis. Specialized pro-resolving mediators (SPMs) have been shown to induce autophagy by downregulating Caspase 2 expression and enhancing cholesterol efflux in THP-1-derived macrophages (Yuan et al., 2025). Lipophagy also facilitates the hydrolysis of cholesterol esters in LDs by modulating neutral cholesterol ester hydrolase (nCEH) and LAL. The liberated free cholesterol is then available for efflux and concurrently activates the LXR-RXR heterodimer, a transcription factor that upregulates ATP-binding cassette transporter A1 (ABCA1) expression and promotes RCT (Jeong et al., 2017b). Additionally, the autophagy substrate protein p62 can activate the mTOR/LXRα signaling pathway, upregulate ABCA1 and ATP-binding cassette transporter G1 (ABCG1) expression, and enhance cholesterol efflux from foam cells (Liang et al., 2019).

### 4.2 Macrophage lipophagy suppresses inflammatory responses

Autophagy has long been recognized as a key regulator of innate immunity and inflammation (Deretic, 2005). ATG16L1 has been demonstrated to be an autophagy-related protein closely associated with the pathogenesis of inflammatory diseases (Cadwell et al., 2008). Atg16L1 deficiency in macrophages can enhance endotoxin-induced interleukin-1β (IL-1β) production through aberrant caspase-1 activation (Saitoh et al., 2008). The interaction between abnormal lipid metabolism and inflammatory pathways due to impaired macrophage lipophagy significantly influences the progression of AS (Chen et al., 2017). When lipophagy is deficient, the accumulation of LDs triggers chronic inflammation (Getiye et al., 2022). Impaired autophagy exacerbates inflammation by skewing macrophage polarization toward the pro-inflammatory M1 phenotype while suppressing the anti-inflammatory M2 phenotype, resulting in elevated secretion of cytokines such as TNF-α and IL-6 (Liu et al., 2015). Moreover, lipophagy promotes triglyceride β-oxidation, increases the production of lipid peroxidation products, inhibits the STING signaling pathway, and reduces the release of pro-inflammatory cytokines (Wang Q. et al., 2024). In both human and mouse atherosclerotic lesions, the expression of LAMP2A is markedly reduced, accompanied by decreased CMA activity in macrophages. This impairment facilitates NLRP3 inflammasome activation and IL-1β secretion, which promotes vascular inflammation and atherosclerotic progression (Qiao et al., 2021).

Emerging evidence indicates that specific microRNAs exert anti-inflammatory and anti-AS effects by modulating macrophage lipophagy. For instance, miR-761 induces autophagy via the mTOR and ULK1 signaling pathways, suppresses foam cell formation in an autophagy-dependent manner, and attenuates the production of pro-atherogenic cytokines to delay atherosclerotic progression (Wang C. et al., 2020). Similarly, miR-100 has been shown to stimulate endothelial autophagy through inhibition of mTORC1, leading to reduced NF-κB activity, mitigated vascular inflammation, and decreased atherosclerotic plaque burden (Pankratz et al., 2018). The miR-33 has been reported to inhibit macrophage autophagy by targeting lipophagy related factors ATG5, ATG7, LAMP1, and LAL (Ouimet et al., 2016). In contrast, anti-miR-33 treatment restores defective autophagy in plaque macrophages, promotes cholesterol efflux and suppresses inflammatory responses, which contributes to reverse AS (Rayner et al., 2011; Ouimet et al., 2017).

### 4.3 Macrophage lipophagy alleviates oxidative stress

During the early stage of AS, ox-LDL is extensively taken up by macrophages via the scavenger receptor CD36 to form foam cells. This process depends on the generation of mitochondrial reactive oxygen species (mtROS). The ox-LDL/CD36 axis reprograms fatty acid metabolism by promoting the transport of long-chain fatty acids (LCFAs) into mitochondria while reducing fatty acid oxidation. This leads to the mitochondrial accumulation of LCFAs, which stimulates mtROS production in macrophages, promotes NF-κB activation and inflammatory responses, and exacerbates AS progression (Chen Y. et al., 2019). Mitochondrial dysfunction exacerbates oxidative stress by elevating intracellular ROS levels, skewing macrophage polarization toward the pro-inflammatory M1 phenotype, and impairing autophagic flux (Hu et al., 2023). The PI3K pathway is critical for autophagic response to ROS level changes by modulating the downstream AKT/mTORC signaling axis. Under moderate ROS levels, both mTORC1 and mTORC2 suppress autophagy, whereas under high ROS conditions mTORC2 exerts the opposite effect (Kma and Baruah, 2022). ROS also enhances lysosomal biogenesis and autophagy by triggering TFEB nuclear translocation (Zhang X. et al., 2016).

Conversely, defective macrophage autophagy aggravates ROS accumulation and inflammatory response, contributing to plaque instability and necrosis. Knockout of the ATG5 in macrophages enhances NADPH oxidase-mediated oxidative stress, increases the apoptosis of macrophages in advanced lesions, and promotes necrotic core expansion (Liao et al., 2012). ATG5 deficiency also shifts macrophage polarization toward the M1 phenotype, exacerbating inflammation and AS (Liu et al., 2015). 7-oxysterol, a major ox-LDL component and pathogenic lipid species abundant in human atherosclerotic plaques, induces cellular lipid accumulation and autophagic dysfunction. Treatment with autophagy inducer rapamycin attenuates oxidative stress, 7-oxysterol-mediated cytotoxicity and lipid accumulation, exerting a protective effect against AS (Yuan et al., 2016).

### 4.4 Macrophage lipophagy attenuates vascular endothelial injury

In the pathogenesis of AS, intercellular communication between macrophages and endothelial cells drives disease advancement by affecting inflammatory cell infiltration, lipid dysregulation, and endothelial damage (Zhang C. et al., 2025). LXR upregulates ABCA1 and ABCG1 expression to promote cholesterol efflux. In the lipid-rich plaque microenvironment, suppression of LXR activity not only induces free cholesterol accumulation and CE remodeling in macrophages, but also enhances the production of pro-inflammatory cytokines and chemokines. These mediators stimulate the expression of endothelial adhesion factors and worsen endothelial inflammation (Leleu et al., 2025). Notably, impaired macrophage lipophagy amplifies this inflammatory cascade by increasing the secretion of IL-1β and enhancing NADPH-mediated oxidative stress and ROS generation (Liao et al., 2012; Sergin et al., 2017). These effects further damage the vascular endothelium and accelerate plaque formation and necrosis.

SIRT6 has been identified as a positive regulator of macrophage autophagy through modulation of the Wnt1/β-catenin pathway (Wang et al., 2023). It reduces the lipid content in foam cells and increases the proportion of vascular smooth muscle cells (VSMCs) by enhancing the autophagy-lysosomal system. Moreover, macrophage autophagy induced by SIRT6 overexpression can inhibit the expression of vascular cell adhesion molecule-1 (VCAM-1), intercellular adhesion molecule-1 (ICAM-1), and P-selectin. This contributes to relieving macrophage infiltration and endothelial cell damage (Wang T. et al., 2020). These are the indirect evidences of macrophage lipophagy on endothelial function, whereas its direct effects in AS remain to be further investigated.

### 4.5 Macrophage lipophagy influences apoptosis

Macrophage autophagy can effectively inhibit ox-LDL-induced endoplasmic reticulum stress and C/EBP homologous protein (CHOP)-mediated apoptosis, thus improving the stability of atherosclerotic plaques. In RAW264.7 macrophages exposed to ox-LDL, cell viability declines, lactate dehydrogenase (LDH) leakage increases, and the apoptotic rate rises. Activation of autophagy attenuates the phosphorylation of inositol-requiring enzyme 1 (IRE1) and the nuclear translocation of activating transcription factor 6 (ATF6). Meanwhile, it upregulates the protein and mRNA expression of glucose-regulated protein 78 (GRP78) and CHOP, which exhibits an inhibitory effect on apoptosis (Tian et al., 2022). Autophagy-dependent apoptosis in foam cells reduces pro-inflammatory cytokine secretion, foam cell proportion, and fatty streak area to improve the outcomes of AS (Wang et al., 2015). Cyclophilin A (CyPA) serves as a key modulator of the crosstalk between macrophage autophagy and apoptosis. By enhancing autophagic activity, CyPA promotes ox-LDL-induced macrophage apoptosis (Xue et al., 2017). Additionally, studies indicate that enhanced autophagy upregulates ABCA1 expression, reduces lipid accumulation in macrophages, and suppresses both foam cell formation and macrophage apoptosis (Zhang B. C. et al., 2016). Induction of macrophage autophagy also protects against 7-oxysterol mediated apoptosis by reducing the permeabilization of lysosomal membrane and oxidative stress (Yuan et al., 2016). See Figure 3.

**FIGURE 3:**
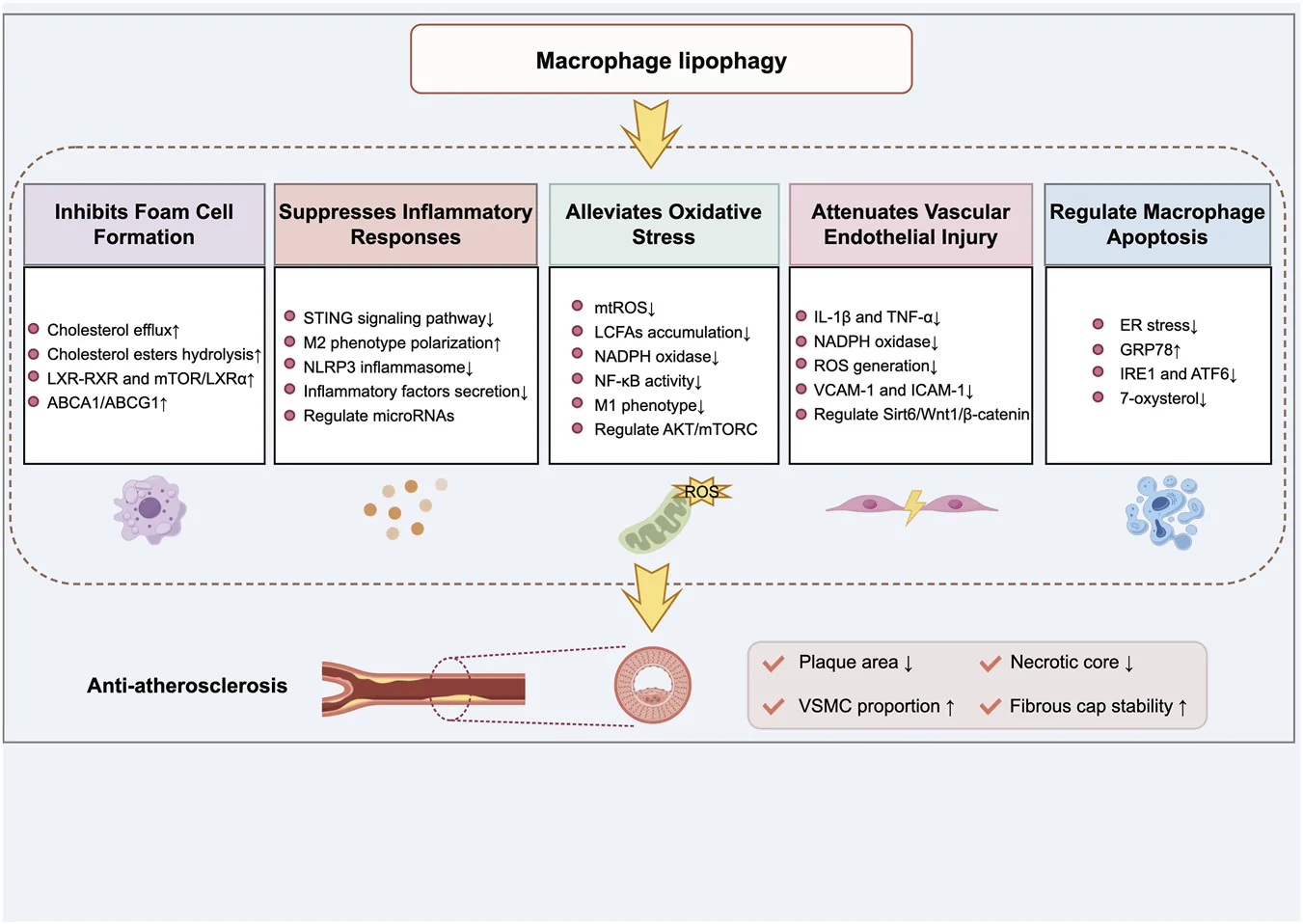
Macrophage lipophagy exerts anti-atherosclerotic effects through multiple pathways, including inhibiting foam cell formation, reducing inflammatory responses, alleviating oxidative stress, attenuating vascular endothelial injury, and regulating apoptosis. These effects collectively reduce plaque area and necrotic core, increase VSMC content, and stabilize the fibrous cap.

## 5 TCM modulates macrophage lipophagy against atherosclerosis

In this section, we focus on the anti-AS effects of TCM modulating macrophage lipophagy. Four categories of agents are discussed: (1) Chinese medicinal metabolites: isolated single bioactive metabolites from TCM herbs; (2) Chinese botanical drugs: dried crude plant materials or drug pairs containing multiple metabolites; (3) Chinese herbal formulas: multi-botanical prescriptions composed according to TCM theory; and (4) CCPPs: manufactured finished products with standardized dosage forms.

### 5.1 Chinese medicinal metabolites

#### 5.1.1 Flavonoids

Calycosin is the major bioactive metabolite and quality evaluation marker of *Astragalus mongholicus* Bunge [Fabaceae; Astragali radix] (Liang et al., 2026). Although calycosin has not been verified to directly regulate lipophagy, it can modulate the lipophagy-related functional molecules and organelles. Mixed lineage kinase domain-like protein (MLKL) is a key regulator of necroptosis, and emerging evidence indicates that it also functions as a novel negative autophagy regulator and transcriptionally repressed by kruppel-like factor 2 (KLF2). Calycosin regulates the KLF2/MLKL pathway to increase Beclin1, ATG5, and LAMP1 levels and reduce p62 accumulation. This promotes LAL-mediated cholesterol hydrolysis and ABCA1-dependent efflux, thereby attenuating foam cell formation, inflammation, and apoptosis in atherosclerotic lesions (Ma et al., 2022). Furthermore, calycosin activates the AMPK/mTOR signaling pathway to initiate autophagy and facilitates autophagosome-lysosome fusion by promoting the formation of the STX17-SNAP29-VAMP8 complex (Zhou et al., 2023).

Luteolin is a flavonoid metabolite found in various medicinal plants. In TCM, it predominantly exists in botanical drugs such as *Lonicera japonica* Thunb. [Caprifoliaceae; Lonicerae japonicae flos] (Zhang J. et al., 2025). It exhibits a range of biological activities, including lipid regulation, anti-inflammatory effects, antioxidant properties, and immunomodulation (Zhu M. et al., 2024). Research suggested that luteolin can decrease intracellular levels of total cholesterol (TC) and CE by activating autophagy, which exhibits increased autophagosome formation, Beclin1 activity, and the ratio of LC3II/I. Enhanced autophagy further promotes cholesterol efflux, inhibits lipid accumulation, and suppresses macrophage apoptosis to alleviate AS (Zhang B. C. et al., 2016; Liao et al., 2018).

Quercetin is widely distributed in various fruits, vegetables, and botanical drugs, which can be used for the prevention of cardiovascular diseases (Cherian et al., 2025). It is the main bioactive metabolite of *Ginkgo biloba* L. [Ginkgoaceae; Ginkgo folium] and *Lysimachia christinae* Hance [Primulaceae; Lysimachiae herba] (Hu et al., 2024; Zhou Y. et al., 2022). In ApoE^−/−^ mice fed with HFD, quercetin increases the expression of mTOR and the LC3II/I ratio, while downregulating the inflammation and senescence markers to alleviate the progression of AS (Cao et al., 2019a). Additionally, quercetin promotes autophagy and delays cellular senescence by suppressing the mammalian sterile 20-like kinase 1 (MST1) signaling pathway *in vitro*, leading to the inhibition of lipid accumulation and foam cell formation (Cao et al., 2019b).

Sixteen compounds were newly identified from *Pueraria montana* var. *lobata* (Willd.) Maesen and S.M.Almeida ex Sanjappa and Predeep [Fabaceae; Puerariae lobatae radix]. Among them, formononetin significantly suppresses the release of inflammatory factors, upregulates the expression of LC3II/I and the M2-macrophage marker CD206, while downregulating the levels of p62 and the M1-macrophage marker CD86. Its anti-inflammatory effect is achieved by enhancing macrophage autophagy and polarization (Xu et al., 2025). See Table 1.

#### 5.1.2 Saponins

Geniposide is the primary bioactive constituent of *Gardenia jasminoides* J. Ellis [Rubiaceae; Gardeniae fructus] and exhibits potential in the prevention and treatment of AS (Jin et al., 2025). Poly (ADP-ribose) (PAR) polymerase 1 (PARP1), traditionally known for DNA damage repair and regulated cell death, has emerged as a regulator of lipophagy. Geniposide inhibits the PARP1/PI3K/AKT signaling pathway to activate lipophagy, which facilitates the interaction between ABCG1 and PLIN1, suppresses foam cell formation of VSMCs, and alleviates AS (Lin et al., 2024b). Moreover, studies revealed that geniposide enhances autophagy activity in ApoE^−/−^ mice by inhibiting phosphorylation of the PI3K/AKT/mTOR pathway, upregulates Beclin1 expression, and downregulates the levels of p62 (Yang et al., 2024). Similar effects also occur in macrophages. The triggering receptor expressed on myeloid cells 2 (TREM2) suppresses autophagy by activating mTOR signaling and inhibiting AMPK and ULK1, leading to decreased levels of the LC3II/I ratio and Beclin1, along with increased p62. Geniposide inhibits the TREM2/mTOR pathway, enhances macrophage autophagy, reduces the size of atherosclerotic plaques and improves plaque stability (Xu et al., 2020).

Gypenoside is derived from the traditional Chinese plants *Gynostemma pentaphyllum* (Thunb.) Makino [Cucurbitaceae; Gynostemmatis herba]. It can regulate the sirtuin 1 (SIRT1)-forkhead box class O1 (FOXO1) pathway, promote the biogenesis of lysosomal, increase the levels of autophagy markers and autophagosome puncta, and dose-dependently restore the autophagic flux impaired by ox-LDL in macrophages. Moreover, it inhibits the uptake of ox-LDL and the transformation of THP-1 cells into foam cells (Hui et al., 2021).

Ginsenoside Rb1 is mainly found in TCM plants including *Panax ginseng* C.A.Mey. [Araliaceae; Ginseng radix et rhizoma] and *Panax notoginseng* (Burkill) F.H.Chen [Araliaceae; Notoginseng radix et rhizoma]. It enhances autophagy by increasing the LC3II/I ratio, promoting the expression of Beclin1 and facilitating the degradation of p62. Meanwhile, it suppresses inflammatory factors and apoptosis to reduce the area of atherosclerotic plaques (Zhou et al., 2018). Moreover, ginsenoside Rb1 promotes AMPK phosphorylation and autophagolysosome formation, which triggers autophagy in macrophage-derived foam cells, inhibits lipid deposition, and stabilizes atherosclerotic plaques (Qiao et al., 2017).

*Celosia argentea* L. [Amaranthaceae; Celosiae semen] is traditionally used in TCM to clear liver heat and improve eyesight. Its active triterpenoid saponin, celosin, can upregulate the expression of LC3, Beclin1, ABCA1 and ABCG1, and downregulate the mRNA expression of CD36 and scavenger receptor-A1 (SR-A1). The anti-atherosclerotic effects are related to promoting macrophage autophagy, inhibit lipid uptake and foam-cell formation, and enhance cholesterol efflux (Tang et al., 2018). Clematichinenoside AR, extracted from *Clematis chinensis* Osbeck [Ranunculaceae; clematidis radix et rhizoma], has similarly been shown to exert anti-atherosclerotic effects by modulating macrophage autophagy. Moreover, it inhibits the activation of NLRP3 inflammasome and inflammatory cytokines (Diao, 2021).

Dipsacoside B is the characteristic constituent of *Lonicera macranthoides* Hand.-Mazz. [Caprifoliaceae; Lonicerae flos]. It has been reported to improve AS by increasing the expression of LC3II, promoting autophagosome formation, inducing autophagy in foam cells, and suppressing macrophage lipid accumulation (Quan et al., 2024).

#### 5.1.3 Alkaloids

Berberine is an alkaloid extracted from traditional Chinese medicines such as *Coptis chinensis* Franch. [Ranunculaceae; Coptidis rhizoma] and *Phellodendron chinense* C.K.Schneid. [Rutaceae; Phellodendri chinensis cortex] (Fan et al., 2025). In recent years, accumulating evidence has indicated that berberine exhibits anti-atherosclerotic effects. Apart from suppressing inflammation and counteracting cellular senescence (Zheng et al., 2025), it also influences macrophage lipophagy related factors. Berberine triggers macrophage autophagy through the AMPK/mTOR signaling pathway and inhibits the secretion of inflammatory cytokines stimulated by ox-LDL (Fan et al., 2015). In a rat model of AS with damp-heat syndrome induced by the high-fat diet under high-temperature and high-humidity conditions, berberine intervention increased the expression of LC3II protein, decreased the expression of p62 protein, and reduced the macrophage foam cell infiltration (Ke et al., 2020). Berberine also promotes lipophagy by modulating the PI3K/AKT/mTOR pathway. It reduces the levels of p62 in a dose-dependent manner, mitigates intimal hyperplasia, promotes the proliferation of carotid artery cells, and inhibits apoptosis (Song and Chen, 2021). These findings have been further validated *in vitro*. Berberine activates SIRT1 to promote TFEB nuclear translocation and deacetylation, which enhances macrophage autophagy and suppresses apoptosis (Zheng et al., 2021). Additionally, it inhibits the RAGE-NF-κB inflammatory signaling axis and simultaneously activates endothelial-protective autophagy (Zhang P. et al., 2025). Indeed, berberine exerts anti-atherosclerotic effects through multiple synergistic mechanisms, including activating macrophage autophagy, inhibiting inflammation, and maintaining vascular endothelial integrity.

#### 5.1.4 Terpenoids

Research has demonstrated that miR-375 mediates the crosstalk between macrophage autophagy and polarization by inhibiting Kruppel-like factor 4 (KLF4). Tanshinone IIA, one of the major bioactive metabolites of *Salvia miltiorrhiza* Bunge [Lamiaceae; Salviae miltiorrhizae radix et rhizoma] (Yin et al., 2025), can regulate the miR-375/KLF4 axis to enhance macrophage autophagy, attenuate lipid accumulation in the aorta, and improve atherosclerotic lesions (Chen W. et al., 2019).

Artemisinin, the characteristic constituent of *Artemisia annua* L. [Asteraceae; Artemisiae annuae herba], promotes macrophage autophagy by modulating the AMPK/mTOR/ULK1 pathway. It mitigates arterial inflammation, inhibits the transformation of macrophages into foam cells, and consequently alleviates atherosclerotic lesions in ApoE^−/−^ mice (Cao Q. et al., 2020). The co-delivery of artemisinin and procyanidins to macrophages within atherosclerotic plaques via biomimetic membrane-coated Prussian blue nanocomplexes promotes the clearance of ROS and NO, and suppresses the NF-κB/NLRP3 signaling pathway. It alleviates inflammation and lipid influx, while simultaneously facilitating cholesterol efflux by activating the AMPK/mTOR-mediated autophagy pathway (Zhou H. et al., 2022).

Ursolic acid, a pentacyclic triterpenoid compound widely distributed in a variety of medicinal plants such as *Prunella vulgaris* L. [Lamiaceae; Prunellae spica] (Mao et al., 2022), retards the progression of AS in mice by activating macrophage autophagy, promoting cholesterol efflux, and attenuating inflammatory responses. It can increase the expression of autophagy-related proteins ATG5 and ATG16L1 while inhibiting IL-1β secretion (Leng et al., 2016).

Ginkgolide C, a key active component of *Ginkgo biloba* L. [Ginkgoaceae; ginkgo folium], enhances the expression of the CMA regulator LAMP2A, improves macrophage autophagic function, suppresses the inflammatory cascade driven by the inflammasome/IL-1β, and ameliorates vascular endothelial dysfunction (Zhang, 2025).

Research suggests that foam cells display lysosomal dysfunction and impaired autophagy. Gastrodin, the characteristic marker component of *Gastrodia elata* Blume [Orchidaceae; Gastrodiae rhizoma], enhances lysosomal biogenesis and autophagic activity via the AMPK-FOXO1-TFEB signaling axis to inhibit macrophage lipid accumulation and inflammatory responses (Tao J. et al., 2021).

#### 5.1.5 Other compounds

Chrysophanol, a characteristic anthraquinone metabolite isolated from *Rheum officinale* Baill. , *Rheum palmatum* L. or *Rheum tanguticum* (Maxim. ex Regel) Balf. [Polygonaceae; Rhei radix et rhizoma], exhibits pharmacological effects such as lipid-regulating and anti-AS (Wang et al., 2022). It enhances the autophagic capacity of macrophages and suppresses foam cell formation by inhibiting the NF-κB/HMGB1-PI3K/AKT/mTOR signaling pathway (Wu et al., 2024).

Laminariae thallus eckloniae thallus, the dried thallus of *Laminaria japonica* Aresch. (Family Laminariaceae), is a typical medicinal and edible homologous substance. Its active metabolite *laminaria japonica* polysaccharide, exerts anti-atherosclerotic effects by activating the SIRT1/FOXO1 signaling axis. This enhances macrophage autophagy, promotes macrophage polarization toward the M2 phenotype, and reduces foam cell formation (Li et al., 2022).

Polydatin, a stilbenoid polyphenol glycoside derived from *Reynoutria japonica* Houtt. [Polygonaceae; Polygoni cuspidati rhizoma et radix], has been shown to promote macrophage autophagy by suppressing the phosphorylation of mTOR protein. Meanwhile, it blocks the assembly and activation of the NLRP3 inflammasome, reduces macrophage apoptosis and the secretion of inflammatory cytokines to inhibit AS (Zhang X. et al., 2023).

Salvianolic acid B, one of the major water-soluble components extracted from *S. miltiorrhiza* Bunge [Lamiaceae; Salviae miltiorrhizae radix et rhizoma], also exhibits protective effects against various cardiovascular diseases. It improves macrophage autophagic dysfunction by inhibiting the AKT/mTOR signaling pathway, reduces the secretion of inflammatory factors, and alleviates apoptosis induced by cholesterol crystals (Sun et al., 2021).

Nevertheless, the literature on isolated metabolites carries some limitations. Most studies test concentrations that exceed physiologically achievable plasma levels without full dose-response curves or determine minimum effective concentrations. Furthermore, these studies largely rely on general autophagy markers rather than assays that directly reflect lipophagy. Future studies should prioritize lipophagy-specific evidence, physiologically relevant dosing, and rigorous pharmacological characterization.

### 5.2 Chinese medicinal plants

The herb pair *Trichosanthes kirilowii* Maxim. [Cucurbitaceae; Trichosanthis fructus] and *Allium macrostemon* Bunge [Amaryllidaceae; Allii macrostemonis bulbus] is commonly employed to relieve chest tightness and heart pain. In LPS-injured macrophages, this herb pair effectively suppresses NLRP3 inflammasome activation and reduces the release of pro-inflammatory factors by enhancing autophagy activity (Bao et al., 2023). Further study suggested that it activates lipophagy in VSMCs by inhibiting the purinergic receptor P2Y12 (P2RY12)/PI3K signaling pathway, reducing lipid accumulation and foam cell formation. Seventeen chemical constituents have been identified, among which albiflorin, apigenin, luteolin, kaempferol, 7,8-dihydroxyflavone, and hesperetin are the major active ingredients responsible for inhibiting P2RY12 (Bao et al., 2024). However, direct evidence that this herb pair induces macrophage lipophagy remains limited and warrants further investigation.

*Salvia miltiorrhiza* Bunge [Lamiaceae; Salviae miltiorrhizae radix et rhizoma] is a widely used Chinese herbal medicine for cardiovascular diseases, known for promoting blood circulation, dispelling blood stasis, dredging meridians, and relieving pain. It also possesses pharmacological activities such as anti-atherosclerotic, anticoagulant, anti-inflammatory, and immunomodulatory effects (Zhao Q. et al., 2026). Extracts of *S. miltiorrhiza* inhibit the proliferation of endothelial cells, induce autophagy in macrophages, enhance lysosomal activity, and suppress the progression of atherosclerotic plaques (Ko et al., 2020).

*Ganoderma lucidum* (Lingzhi) refers to the dried fruiting body of *G. lucidum* (Leyss. ex Fr.) Karst. or *Ganoderma sinense*, fungi belonging to the Polyporaceae family. Traditionally, it is used to replenish Qi, soothe the mind, relieve cough and wheezing, and offer nourishment and tonification. Notably, Ganoderma exhibits anti-atherosclerotic activity by modulating glucose-lipid metabolism and the gut microbiota (Ma et al., 2024). *Ganoderma lucidum* spore powder has been reported to reduce the plaque area and aortic calcification in mice at both early and late stages of AS. It can inhibit lipid deposition and apoptosis, decrease the size of necrotic core, increase the collagen content, and enhance the stability of atherosclerotic plaque. Importantly, it suppresses foam cell formation by enhancing cholesterol efflux mediated by ABCA1 and ABCG1 in macrophages (Zheng et al., 2023). These effects are closely associated with macrophage lipophagy, and the underlying mechanisms remain to be further explored. Table 2 summarizes the Chinese herbal medicines that regulate macrophage lipophagy related molecules against AS.

For botanical drug extracts, insufficient chemical characterization further weakens the evidence. Extraction solvent, drug-to-extract ratio, and chemical fingerprinting are often omitted, and the active metabolites driving autophagy modulation are rarely identified.

### 5.3 Chinese herbal formulas

Huanglian Jiedu Decoction, recorded in *Zhouhou Beiji Fang*, is a classic traditional Chinese herbal formula well-known for its heat-clearing and detoxifying properties. The decoction contains active substances, including geniposide, baicalin, coptisine, epiberberine, berberine, xanthine, and palmatine. It has been demonstrated to enhance the efferocytosis of apoptotic cells through macrophages, and improve the stability of atherosclerotic plaques (Zhou et al., 2024). Furthermore, it can inhibit the P2RY12/PI3K/AKT signaling pathway, activate autophagy, and suppress the formation of foam cells (Lin et al., 2024a).

Zhishi Xiebai Guizhi Decoction, a classic prescription documented in Zhang Zhongjing’s *Jin Gui Yao Lue* (Synopsis of the Golden Chamber), is traditionally used to treat chest impediment caused by phlegm and qi stagnation. It exhibits anti-inflammatory, antioxidant, lipid regulating, and vascular endothelial-protective effects (Mo et al., 2025). This decoction has been shown to reduce the area of atherosclerotic plaques in mice. An integrated analysis employing UPLC-Q-TOF-MS, network pharmacology, and molecular docking suggests that it coordinately activates autophagy and inhibits apoptosis by modulating the AGE/RAGE signaling pathway, thus attenuating inflammation and slowing the progression of atherosclerotic plaques. This effect was validated in animal models (Zhang Z. et al., 2024).

Taoren Honghua Decoction, the active ingredients of which are quercetin, apigenin, luteolin, kaempferol, and tanshinone IIA, can reduce lipid accumulation and plaque formation. It activates lipophagy by inhibiting the PI3K/AKT signaling pathway, promotes the expression of cholesterol efflux proteins ABCA1 and ABCG1, and suppresses inflammatory responses (Chang et al., 2025).

Several empirical formulas have also been demonstrated to ameliorate AS by regulating lipophagy-related molecules. Tiaozhi Tongmai Granules is composed of *Astragali Radix*, *Angelicae Sinensis Radix*, *Crataegi Fructus*, and other herbs. It can promote the co-localization of autophagosomes, lysosomes, and LDs, enhance autophagic flux and autolysosomal degradation capacity, and reduce the area of aortic lipid plaques (Zheng et al., 2015). Sanjie Tongmai Formula has been reported to inhibit the PI3K/AKT/mTOR signaling pathway, promote macrophage M2 polarization and autophagy, regulate lipid metabolism, suppress inflammatory responses, and alleviate AS plaque injury (Li et al., 2023). Bushen Jiangzhi Formula can improve the atherosclerotic lesions induced by high-fat-diet in mice, regulate lipid metabolism, enhance autophagy, and inhibit inflammation (Cao et al., 2020). Bushen Kangshuai Tablets can activate autophagy in macrophages by inhibiting the PI3K/AKT/mTOR pathway and promote the transition from M1 to M2 phenotype via the PPARγ/NF-κB axis. Consequently, they can synergistically suppress inflammation and slow down the progression of AS (Xie et al., 2020).

The Huayu Qutan Formula, whose core active components include ferulic acid, chlorogenic acid, calycosin, formononetin, among others, exerts anti-AS effects through a dual-action mechanism. On one hand, it targets the mTOR/TFEB axis to activate lipophagy, increases the number of autolysosomes and lysosomes, and enhances LD degradation in macrophages. On the other hand, it specifically upregulates the expression of cholesterol efflux proteins ABCA1 and SCARB1, synergistically reducing the formation of foam cells (Li et al., 2025; Li et al., 2024). Mass spectrometry analysis has identified taurine as a key active constituent of this formula, likely serving as the critical compound mediating the anti-atherosclerotic effects through the mTOR/TFEB lipophagy pathway (Li et al., 2024). See Table 3.

### 5.4 Commercial Chinese polyherbal preparations (CCPPs)

Danlou Tablets have been reported to activate macrophage autophagy by inhibiting the PI3K/AKT/mTOR signaling pathway. It inhibits lipid deposition, foam cell formation, macrophage infiltration and inflammatory responses, thus ameliorating atherosclerotic lesions. These effects have been verified in both animal models and cellular experiments (Liu C. et al., 2021).

Liquid chromatography- tandem mass spectrometry was used to identify the chemical constituents of Sanwei Tanxiang Capsules, and network pharmacology was applied to predict the potential targets. The results suggested that this formulation can promote cholesterol efflux, lower blood lipid levels, and inhibit the formation of atherosclerotic plaques by enhancing lipid uptake and lysosomal activity in adipose tissue macrophages (Ye et al., 2023).

Shenyuan Dan Capsules can activate macrophage autophagy by inhibiting the PI3K/AKT/mTORC1 pathway. It increases Beclin1 expression and the LC3II/I ratio, while reducing TC, free cholesterol (FC), and CE levels to suppress foam cell formation and cholesterol esterification. It also improves blood lipid profiles, decreases aortic root plaque area, and lowers the AS index in mice (Zhou et al., 2019). Similarly, Tianxiang Dan inhibits the PI3K/AKT/mTOR pathway to activate macrophage lipophagy. It upregulates the expression of TFEB, LC3II/I, and ULK1, downregulates p62, and increases the cholesterol efflux protein ABCA1. These actions synergistically reduce lipid deposition and exert anti-atherosclerotic effects (Zhang Y. et al., 2025).

Tongxinluo can enhance Beclin1-mediated autophagy, facilitate the dissociation of the Beclin1 and Bcl-2 complex, and inhibit macrophage apoptosis to reduce the vulnerability of advanced atherosclerotic plaques (Chen et al., 2018). Moreover, Tongxinluo downregulates the expression of histone deacetylases (HDACs), upregulates the protein levels of LC3II, Beclin1, ABCA1, and ABCG1, reduces the expression of LOX-1 protein, enhances macrophage lipophagy, and suppresses lipid deposition and the formation of foam cells (Chen et al., 2021). See Figure 4 and Table 4.

**FIGURE 4:**
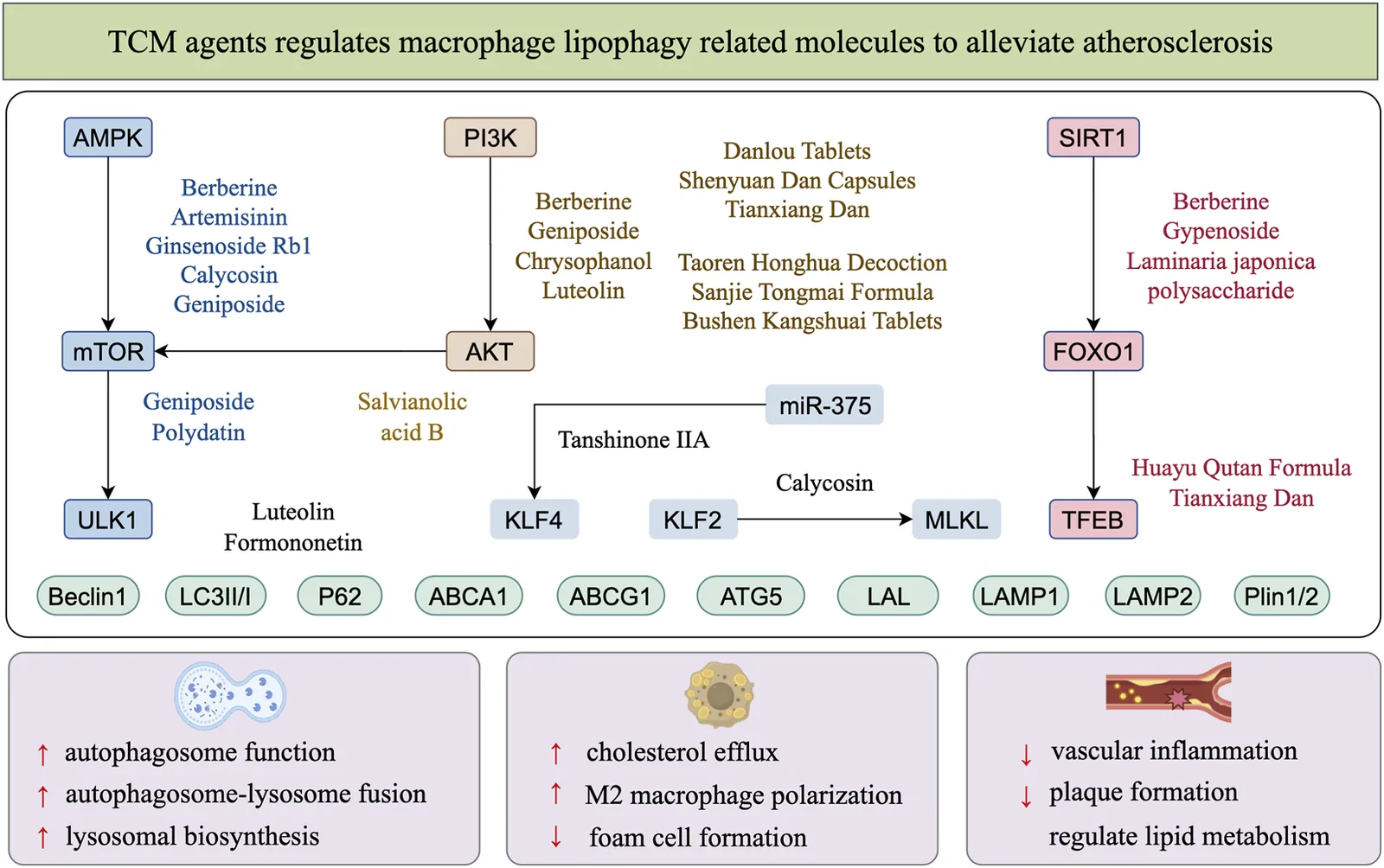
TCM agents—including medicinal metabolites, herbal formulas, and CCPPs—regulate macrophage lipophagy via three core signaling axes (AMPK/mTOR/ULK1, PI3K/AKT, and SIRT1/FOXO1/TFEB) and transcriptional regulators, converging on lipophagy effector molecules to enhance autophagic flux and cholesterol efflux, suppress foam cell formation and vascular inflammation, and alleviate AS.

## 6 Conclusions and perspectives

In this review, we have systematically synthesized current evidence on the interplay between macrophage lipophagy and AS, as well as the therapeutic potential of TCM-derived interventions. Macrophages degrade LDs through three distinct lipophagic pathways—macrolipophagy, microlipophagy, and CMA-mediated lipophagy—each converging on the lysosomal compartment for terminal lipid hydrolysis. Dysregulation of these pathways promotes foam cell formation, sustains pro-inflammatory polarization, and accelerates atherosclerotic lesion progression. These three types of lipophagy are not independent but rather interact with one another; however, their interrelationships remain underexplored and warrant further investigation. Whether intermediary factors, crosstalk mechanisms, or other underlying processes modulate the relationship between macrophage lipophagy and AS requires further study.

Furthermore, we have summarized how TCM—including active metabolites, medicinal plants, herbal formulas, and CCPPs—counteracts the pathological processes of AS by restoring or enhancing macrophage lipophagic flux. Their mechanisms of action converge on several core aspects of lipophagy regulation: 1) Modulation of key lipophagic signaling nodes. Multiple active TCM ingredients regulate central lipophagic signaling pathways, notably the AMPK/mTOR and PI3K/AKT axes, which promote autophagosome formation and enhance macrophage lipophagy. 2) Facilitation of LD degradation and autophagosome-lysosome fusion. TCM components upregulate the expression of autophagy-related proteins and facilitate the assembly of key complexes to promote the precise recognition and targeting of LDs. Moreover, they induce the fusion between autophagosomes and lysosomes to activate lipophagy. 3) Regulation of cholesterol efflux and inflammation. TCM enhances lipophagic degradation while simultaneously promoting cholesterol efflux, suppressing the NLRP3 inflammasome and driving macrophage M2 polarization to inhibit lipid accumulation and plaque progression. 4) Restoration of organellar homeostasis. Beyond simple lipid degradation, TCM also protects mitochondrial function and promotes lysosomal biogenesis, addressing the organellar dysfunction that underlies advanced AS.

While evidence supporting the role of TCM in regulating macrophage lipophagy is encouraging, several challenges remain. Although the monomeric compounds highlighted in this review offer potential leads for new drug development, herbal formulas represent the core of TCM clinical practice. The precise bioactive metabolites, pharmacokinetics, and molecular targets of these complex formulas remain largely unexplored. The key challenges are as follows: 1) Insufficient specificity: Most TCM-derived metabolites induce general autophagy rather than macrophage-specific lipophagy, and selective lipophagy inducers are scarce. 2) Off-target risks: Autophagy is essential for all cell types, and its systemic activation may exert unpredictable effects on other cells or tissues. 3) Stage dependency: Autophagy may be protective in early AS but may promote plaque cell survival and necrotic core expansion in advanced lesions, necessitating stage-specific strategies. 4) Inadequate standardization: Chinese herbal formulas and CCPPs show substantial batch-to-batch variability, and identification of active constituents remains challenging. 5) Lack of clinical evidence: Most data are derived from *in vitro* and animal studies; clinical studies directly measuring macrophage lipophagy biomarkers and cardiovascular endpoints are scarce.

Future research should employ well-designed randomized controlled trials to evaluate the impact of TCM interventions on macrophage lipophagy, atherosclerotic plaque burden, and cardiovascular events. Additionally, the application of systems biology approaches—such as multi-omics analysis—combined with advanced cell biology techniques like live-cell imaging and CRISPR-based screening, will be essential for mapping the comprehensive interaction networks between TCM components and the macrophage lipophagic machinery. These efforts will provide a stronger foundation for subsequent clinical translation and the development of novel therapeutic agents.
